# ATP-independent entropy-driven dsRNA unwinding by DDX3X revealed by coarse-grained simulations and deep learning

**DOI:** 10.1371/journal.pcbi.1014928

**Published:** 2026-10-30

**Authors:** Kang Wang, Chun-Lai Ren, Yu-Qiang Ma

**Affiliations:** 1 National Laboratory of Solid State Microstructures and Department of Physics, Collaborative Innovation Center of Advanced Microstructures, Nanjing University, Nanjing, China; 2 Hefei National Laboratory, Hefei, China; University of Missouri, UNITED STATES OF AMERICA

## Abstract

DEAD-box RNA helicases (DDXs) are traditionally known as ATP-dependent motors that unwind double-stranded RNA (dsRNA). Recent experiments, however, show that some DDXs promote dsRNA unwinding even in the absence of ATP, raising a fundamental question about the physical mechanism underlying ATP-independent strand separation. Here, we develop a minimal, physics-based coarse-grained RNA model and incorporate weak, specific interactions between DDX3X and dsRNA, revealing the inherently stochastic nature of unwinding events. The unwinding process must overcome an energy barrier, but thermal fluctuations and entropy gain provide a driving force for RNA remodeling. We identify that dsRNA separation proceeds through rare yet obligatory strand-displacing intermediates facilitated by DDX3X. By combining deep learning-assisted analysis, we further rank the contributions of different entropic components, revealing hydrogen bonding as the dominant factor, followed by base stacking and then the backbone conformation. These findings reveal a previously unrecognized physical mechanism for RNA duplex unwinding and offer an effective framework for studying RNA remodeling kinetics.

## Author summary

Molecular motors are generally thought to require chemical energy to perform mechanical work. ATP-dependent helicases are a classic example: they use ATP to separate nucleic-acid strands. Recent experiments, however, have challenged this conventional view by showing that DDX3X can promote strand separation without ATP. This raises a fundamental question: if no chemical energy is consumed, what drives the strands apart? We used computer simulations to address this question for DDX3X-mediated dsRNA unwinding in the absence of ATP. We found that strand separation is associated with RNA remodeling. Most fluctuations do not lead to complete separation, but rare fluctuations can drive the RNA from the paired state to an unwound state. We further used machine learning to identify which molecular interactions contribute most strongly to this process. Our results suggest that some molecular machines can promote structural remodeling even without direct chemical energy input, by exploiting the thermal fluctuations and entropy inherent to the molecular system. This provides a possible physical mechanism for ATP-independent activity and broadens our understanding of how molecular machines can function.

## Introduction

DEAD-box helicases (DDXs), one of the largest and most evolutionarily conserved families of RNA helicases, are present in all kingdoms of life [1]. A representative member of this family, DDX3X, is an ATP-dependent RNA helicase that regulates multiple aspects of RNA metabolism, including transcription [2], splicing [3], transport [4], translation [5], and degradation [6]. Given that many RNA metabolic processes require the establishment of specific RNA structures, the helicase core of DDX3X—comprising its two RecA-like structural domains (D1 and D2)—unwinds short double-stranded RNA (dsRNA) into single-stranded RNA (ssRNA) [7]. Dysfunctional variants of DDX3X are etiologically linked to multiple human pathologies, most notably the neurodevelopmental disorder termed DDX3X syndrome [8]. A recent study by Owens et al. [9] demonstrated that pathogenic mutations in DDX3X compromise RNA unwinding capacity and translational efficiency, suggesting a plausible mechanism underlying these human pathologies.

The central role of DDX3X in cellular homeostasis has spurred extensive studies on its RNA remodeling mechanism. Song and Ji [7] showed that two DDX3Xs cooperatively unwind a 23-bp dsRNA: each domain binds ssRNA, with conformational changes triggered by ATP that allow unwinding. Toyama et al. [10] further demonstrated the stronger affinity of DDX3X for ssRNA over structured motifs, underpinning its strand separation activity. DDXs thus employ a localized strand separation mechanism that involves direct duplex binding and ATP-dependent melting, different from the processive translocation seen in DNA helicases [11]. Strikingly, Yanas et al. reported that DDX3X unwinds structured RNAs without ATP [12], raising a fundamental question: how does ATP-independent unwinding occur? This mechanistic puzzle presents substantial experimental hurdles, underscoring the need for effective coarse-grained models of RNA-protein complexes.

Current RNA models, ranging from simplified semi-flexible chain representations [13] to more detailed Martini3 [14] and oxRNA [15] models, struggle to effectively simulate RNA unwinding process due to insufficient resolution or limitations in simulation timescales. To bridge this gap, we developed HelixTriad coarse-grained RNA model by adopting the three-bead-per-nucleotide framework proposed in previous studies [16–18], where base-stacking and hydrogen‑bonding interactions are physically decoupled via distinct base and hydrogen‑bonding beads. It effectively captures the conformational transitions observed experimentally during dsRNA melting, quantitatively reproduces experimental melting temperatures. It also yields persistence lengths consistent with experimental measures and reproduces the semiflexible conformations of dsRNA. Furthermore, this model can be extended to RNA-protein complexes. Using Langevin dynamics simulations, we investigated both DDX3X-dsRNA binding and unwinding dynamics. Our results reveal that dsRNA unwinding occurs stochastically and is driven by entropy gains under thermal fluctuations, a mechanism closely mirroring the canonical DNA denaturation model proposed by Jensen and Hippel in 1976 [19]. Notably, successful unwinding trajectories involve crossing an energy barrier that arises from the competition between enthalpy and entropy. Given that entropy is inherently unmeasurable, we turned to machine learning to identify the key factors governing its change. We developed Entropy-UNet and demonstrated its capability to predict entropy variations during the unwinding of dsRNA under the influence of DDX3X. Furthermore, this method can dissect the distinct contributions of various interactions within the complex system to the overall entropy change, providing new insights into the fundamental nature of entropy.

## Results

### Development and validation of the HTri–CG model

The three-bead-per-nucleotide framework has a long history. Hyeon and Thirumalai introduced explicit base stacking into a three-bead RNA model [23]. Denesyuk and Thirumalai subsequently added an explicit directional hydrogen-bonding potential, but both stacking and hydrogen bonding acted on the same base bead and thus could not be physically decoupled [24]. Recently, Kapoor, Kim, and Mittal adopted a three‑bead DNA representation with distinct phosphate‑sugar, base, and hydrogen‑bonding sites [16]. Building on this idea, we built upon a three‑bead‑per‑nucleotide framework, and parameterized three distinct interaction sites to achieve physical separation of hydrogen bonding, base stacking and phosphodiester backbone electrostatics, yielding HelixTriad coarse-grained (HTri–CG) RNA model (Fig 1a). In this representation, each nucleotide is decomposed into three physically distinct beads: a sugar‑phosphate backbone bead responsible for backbone electrostatics, a base bead for base‑stacking interactions, and a dedicated hydrogen‑bond bead that mediates Watson‑Crick (WC) base‑pairing. This three‑bead design enables physical decoupling of backbone electrostatic, base‑stacking, and hydrogen‑bonding interactions, rather than lumping multiple chemical contributions into a single bead. All bead types, interaction potentials, and full parameter sets are comprehensively documented in the Coarse–Grained Modelling of S1 Appendix.

**Fig 1:**
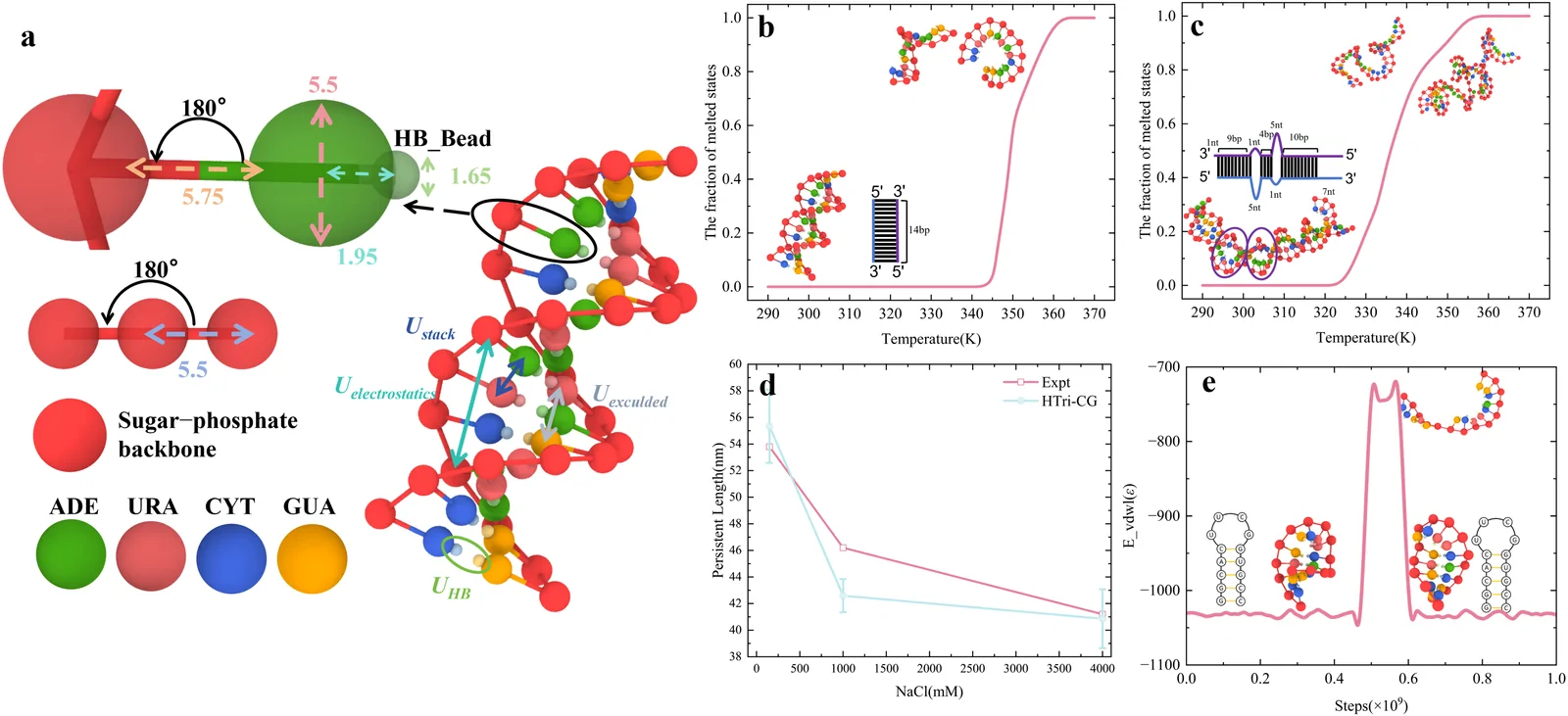
Development of HTri-CG RNA model and predication of dsRNA melting curves. (a) Schematic diagram of the HTri–CG RNA model architecture. Units: see Supplementary Information’s Length-scale mapping. (b)–(c) Thermodynamic characterization of dsRNA denaturation (28-nt S1S2 and 73-nt XY) using REMD. The fraction of melted states is plotted as a function of temperature (290–370 K). Representative local configurations are shown for each dsRNA system. References for the experiment are sourced from Serra [20] and Wu [21]. The purple circle in (c) highlights two interloop structures in the XY duplex. (d) The persistence lengths of 20-bp dsRNA from equilibrated MD trajectories at three NaCl concentrations (150 mM, 1000 mM, and 4000 mM). The experiment is referenced from Dong [22]. (e) Time-dependent evolution of vdW interaction energies at 300K, with insets illustrating three-dimensional hairpin conformations and corresponding secondary structure diagrams sampled at three distinct temporal phases.

In vitro studies have shown that DDX3X can bind and unwind 13–19 bp dsRNA [25]. To validate the HTri-CG RNA model, we characterized the melting curve and flexibility of two dsRNA duplexes (see section of dsRNA and Hairpin constructs in S1 Appendix): a 14-bp (28-nt) duplex composed of strands S1 and S2 and a 23-bp (73-nt) duplex consisting of strands X and Y. The simulation box was set to 30 x 30 x 30 nm^3^ and contained ten dsRNA molecules to ensure statistical reliability. Replica exchange molecular dynamics (REMD, see Replica exchange molecular dynamics Implementation in S1 Appendix) simulations were employed to enhance sampling efficiency. Melting curves were obtained by statistically analyzing simulation snapshots to distinguish between hybridized and melted (≥60% HB broken) states, as defined in Fig 1b-1c. Both the 14-bp S1S2 and 23-bp XY dsRNAs exhibited three distinct regimes: (1) Stable Hybridization (290–345 K for S1S2; 290–325 K for XY): The duplexes maintained stable hybridized states. (2) Partial Melting (345–359 K for S1S2; 325–355 K for XY): A coexistence of hybridized and melted states was observed. (3) Complete Melting (≥359 K for S1S2; ≥ 355 K for XY): The fully melted state became dominant. The melting temperature (*T*_*m*_), corresponding to 50% population of melted states, was determined to be 349.5 ± 0.3 K for S1S2 (experimental reference value *T*_*exp*_ = 349.4 K [20]) and 336.5 ± 1.0 K for XY (*T*_*exp*_ = 337.4 ± 0.5 K [21]). Given that the experimental melting curves were measured under dilute-solution conditions, we reduced the number of dsRNA molecules in the system from ten to six. The resulting melting curves remained essentially unchanged (Fig A in S1 Appendix).

Our structural analysis revealed two predominant conformations of ssRNA in the melted state: single-helical strands and open-ring structures (Fig 1b-1c). The open-ring configuration exhibited remarkable persistence mediated by base-stacking interactions, which are known from our experience to predispose such structures toward hairpin formation during kinetic processes. This observation prompted validation of the semi-flexibility parameterization in our coarse-grained RNA model. We calculated the bending persistence length (P) of 20-bp dsRNA from equilibrated MD trajectories at three NaCl concentrations(150 mM, 1000 mM, and 4000 mM), following Dong et al. [22]. For each concentration condition, 300 fragments of 13-bp (L*c*, θ < 30°) dsRNA (excluding three terminal base pairs) were randomly sampled; bending angles were binned at 0.5°, and the distribution *p(θ)* was fitted to the equation $\;-ln(p(\theta )/sin\theta )=P\theta^{\text{2}}/\text{2}L_{c}$, yielding P values in good agreement with experiments (Fig 1d). To further validate the model for short ssRNA, we performed extensive MD simulations (total 1 × 10^9^ steps) starting from a folded 14-nt tetraloop hairpin with a 5-bp stem (Fig 1e). While the hairpin is energetically favored from a van der Waals (vdW) perspective (including base stacking, hydrogen bonding, and steric effects), consistent with Nozinovic et al. [26], it transiently unfolds into an open-ring structure and refolds within the duration of the simulation. This reversible transition confirms that the semi-flexibility parameters of our HTri-CG RNA model are well calibrated. Additionally, Table A in S1 Appendix tabulates ideal A-RNA structural data for comparison, with our HTri–CG RNA exhibiting favorable consistency. Overall, those results that are in close agreement with experimental melting temperatures, persistence lengths, and the diverse RNA conformations observed further confirm that the HTri-CG Model reliably captures RNA conformational dynamics and is suited for investigating short dsRNA unwinding.

### Intrinsic Stochasticity of ATP-independent dsRNA unwinding by DDX3X

With the introduction of DDX3X, an important issue is describing the interactions between DDX3X and dsRNA. Previous work has established that their interactions are primarily mediated by specific recognition between RecA-like D1/D2 domains and RNA nucleotide phosphate groups and sugar moieties [7]. To quantify the binding specificity, we integrated a sticker-and-spacer model [27]. The binding affinity was determined using a systematic titration approach. We progressively increased the interaction strength between the D1/D2 domains and the Sugar Phosphate Bead (SPB) in increments of 0.72 ε (where ε is the binding energy in kcal/mol) until observable dsRNA duplex unwinding events were detected. Such unwinding events are stochastic and restricted to a narrow window of attraction strengths. Both insufficient and excessive interactions suppress strand separation. This procedure yielded a working binding energy parameter of 10.00 ε for dsRNA-DDX3X D1/D2 interaction. All coarse-grained simulations were performed in the NVT ensemble using a Langevin thermostat at room temperature. Each simulation system consisted of a single DDX3X and one 14-bp dsRNA molecule placed in a cubic box with periodic boundary conditions. To mimic a dilute solution and assess finite-size effects, we used two different box sizes: 15 x 15 x 15 nm^3^ and 30 x 30 x 30 nm^3^. For each box size, 50 independent replicas were conducted. The simulations were preformed using the LAMMPS software package [28]. Random initial trials yielded diverse outcomes within the simulation duration, which we categorized into four distinct states based on the observed unwinding extent of the dsRNA:

(i). Stable Bound (SB): The dsRNA remained fully base-paired and stably complexed with DDX3X.
(ii). Partially Unwound (PU): DDX3X partially disrupted the dsRNA duplex, leading to localized strand separation.
(iii). Fully Unwound (FU): DDX3X completely dissociated the dsRNA into two single-stranded RNAs.
(iv). Partially Re-closed (PR): A special category of the PU state. This state formed from a FU via subsequent re-annealing of the separated strands, resulting in a partially paired structure.

The existence of four distinct conformational states implies that dsRNA unwinding is a stochastic process. To quantify the unwinding efficiency, we calculated the dwell probabilities of these final states, averaged over 50 independent replicas, shown in Fig 2a. Due to the interconversion between the FU and PR states, they were analyzed as a combined state (FU&PR). In both simulation box sizes, successful unwinding events were rare, with a low transition probability of 0.18 for the complete unwinding pathway SB → PU → FU. Among the four states, PU is the most probable. Given that dsRNA unwinding involves multiple steps of conformational change, we further calculated the probability of each transition step. These probabilities, referred to as transition rates, are presented in Fig 2b. Notably, the transition PU → FU has the lowest probability, indicating that PU is the primary kinetic bottleneck in the unwinding process. In addition, because dsRNA first binds to DDX3X before unwinding, we defined the time from the start of the simulation to the onset of unwinding as the pre-unwinding duration. In Fig 2c, the x-axis represents the pre-unwinding duration, while the y-axis indicates the number of simulations in which no conformational transition had occurred by that time. These resulting distributions exhibit Poisson-like characteristics, supporting that the onset of unwinding is stochastic.

**Fig 2:**
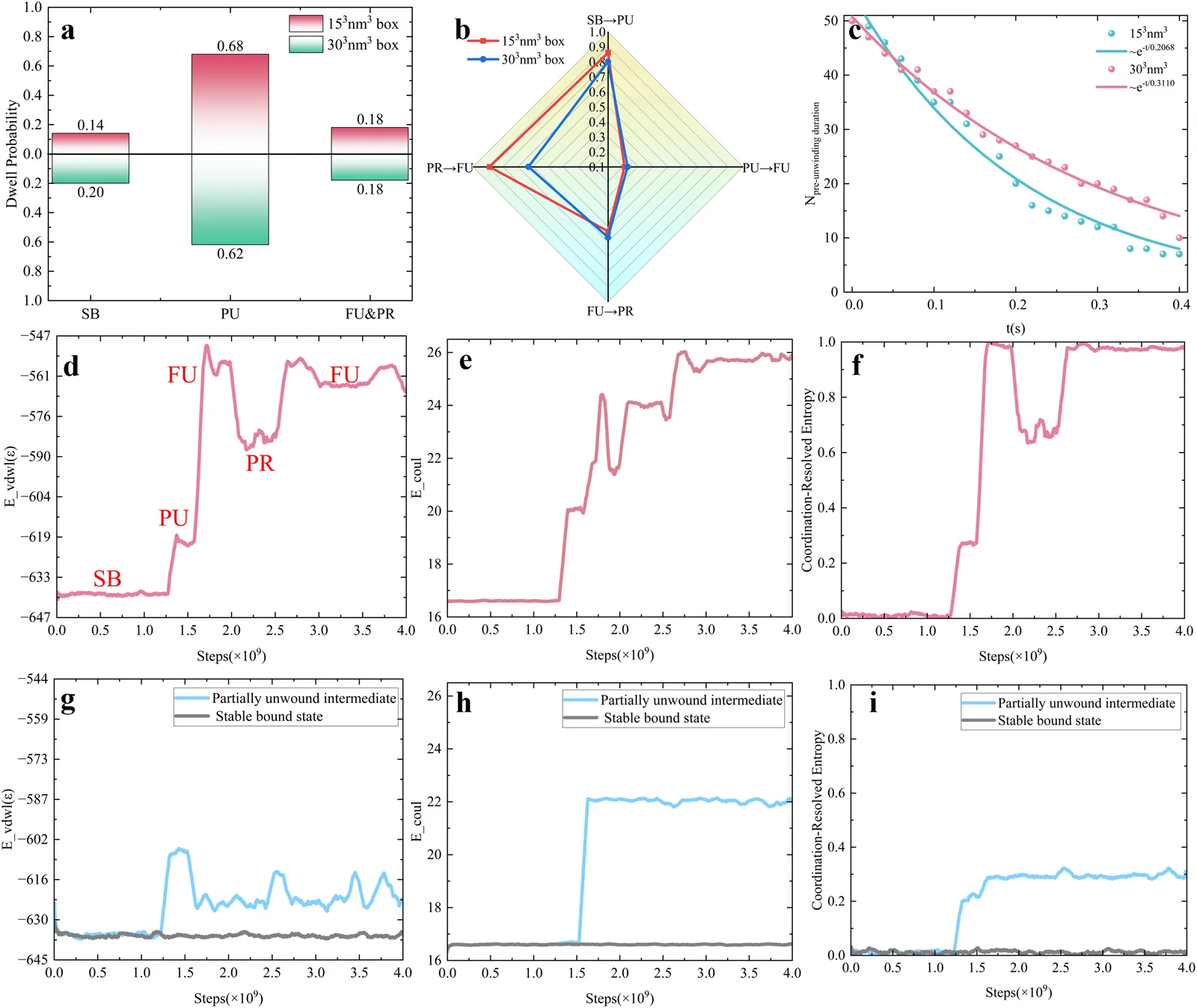
Stochastic, entropy-driven dsRNA unwinding mediated by DDX3X. (a) State dwell probabilities (SB, PU, FU&PR); (b) population of transition among the four transition pathways (SB → PU, PU → FU, FU → PR, PR → FU); and (c) distributions of pre-unwinding durations, averaged over 50 independent replicas for two box sizes. (d)∼(f) and (g)∼(i) present the analyses of vdW, Coulombic forces, and CRE for one successful unwinding event and for two abortive unwinding attempts, respectively, conducted in the 15 x 15 x 15 nm^3^ simulation system.

It’s noteworthy that the SB state demonstrates RNA clamping functionality, characterized by prolonged RNA binding capacity [29]. This observation aligns with established experimental evidence demonstrating that DDXs can form stable, long-lived RNA complexes [30]. The structure of the PU conformation is investigated by Toyama et al., using ^19^F probe at 2023 [10], identifying it as a transient intermediate during RNA unwinding. Furthermore. PU involved 4–8 bp disruptions in 100 independent MDs, revealing the variable nature of this intermediate state. The PR state exhibits unstable unwinding activity of DDX3X, requiring persistent disruption of the energy barriers maintained by base stacking and hydrogen bonding interactions. Specifically, this destabilization predominantly occurs at terminal base pairs (2–3 bp), revealing position-dependent stability patterns during duplex reformation.

### Entropy-driven dsRNA unwinding by DDX3X

To investigate the primary driving mechanism behind the successful unwinding of dsRNA, we examined the system’s enthalpic and entropic contributions to elucidate their respective roles. Coordination-resolved entropy (CRE; see Materials and Methods) effectively captures the conformational changes accompanying double-strand separation. Fig 2d-2f depict the temporal evolution of vdW interactions, Coulombic potentials, and CRE changes during a successful unwinding event, which proceeds along a specific pathway: SB → PU → FU → PR → FU. As the bases separate, vdw attractions weaken, whereas preferential binding of DDX3X to the ssRNA backbone brings RNA backbone beads into closer proximity, thereby strengthening Coulombic interactions. The resultant net positive enthalpy change (∆H > 0) thermodynamically disfavors spontaneous unwinding. However, progressive entropy gains (∆S > 0) across successive states (SB → PU → FU → PR → FU) overcome the enthalpic barrier, confirming the process as entropy-driven. The entropy compensation mechanism originates from increased conformational freedom in ssRNA strands.

Although successful unwinding can occur, unsuccessful cases still abundantly exist: transitions from the SB to PU states that result in persistent PU state, as well as prolonged stability in the SB state. Fig 2g-2i illustrates these dynamics, with sky blue curves representing PU state transitions and gray curves denoting SB state behavior. Notably, the PU state (sky blue) demonstrates a characteristic vdW interaction surge, beginning around ~1.25 × 10^9^ steps. Structural analysis of snapshots reveals concurrent disruption of 3-bp during this stage. Subsequently, the number of disrupted base pairs fluctuates before stabilizing, leaving only eight base pairs intact in the PU state. Coulomb interactions exhibited minimal fluctuation, following a step-like behavior corresponding to the sequential opening of base pairs. CRE effectively tracked this progressive process with high sensitivity. In contrast, the SB state (gray) maintains stable across all parameters (vdW, coulomb interactions, and entropy), keeping the RNA clamping throughout the process—a futile unwinding attempt during the unwinding cycle.

### Unwinding dynamics and energy barrier of DDX3X-dsRNA complex

To explore the unwinding dynamics, we employed *Distance* and *Rg*, two key metrics derived from MD trajectories. *Distance* represents the center-of-mass distance between the two strands of dsRNA, while *Rg* quantifies the overall compactness of the DDX3X-dsRNA complex. A representative successful unwinding trajectory was chosen to illustrate the conformational dynamics during the process. Fig 3a-3b show the evolutions of *Distance* and *Rg*, with several representative states (I-VII) highlighted along the trajectory. For clarity in visualizing structural transitions, the intrinsically disordered N-terminal region (IDR) of DDX3X was omitted in Fig 3c.I-VII, as it was not associated with the dsRNA during the unwinding process (Fig B in S1 Appendix). Consistent with our observations, previous studies have also reported minimal IDR binding to poly-U_10_ ssRNA, GC-14mer dsRNA, and a 14mer tetraloop RNA [31]. The system follows a sequence of conformational transitions: I → II → III → IV → V → VI → VII.

**Fig 3:**
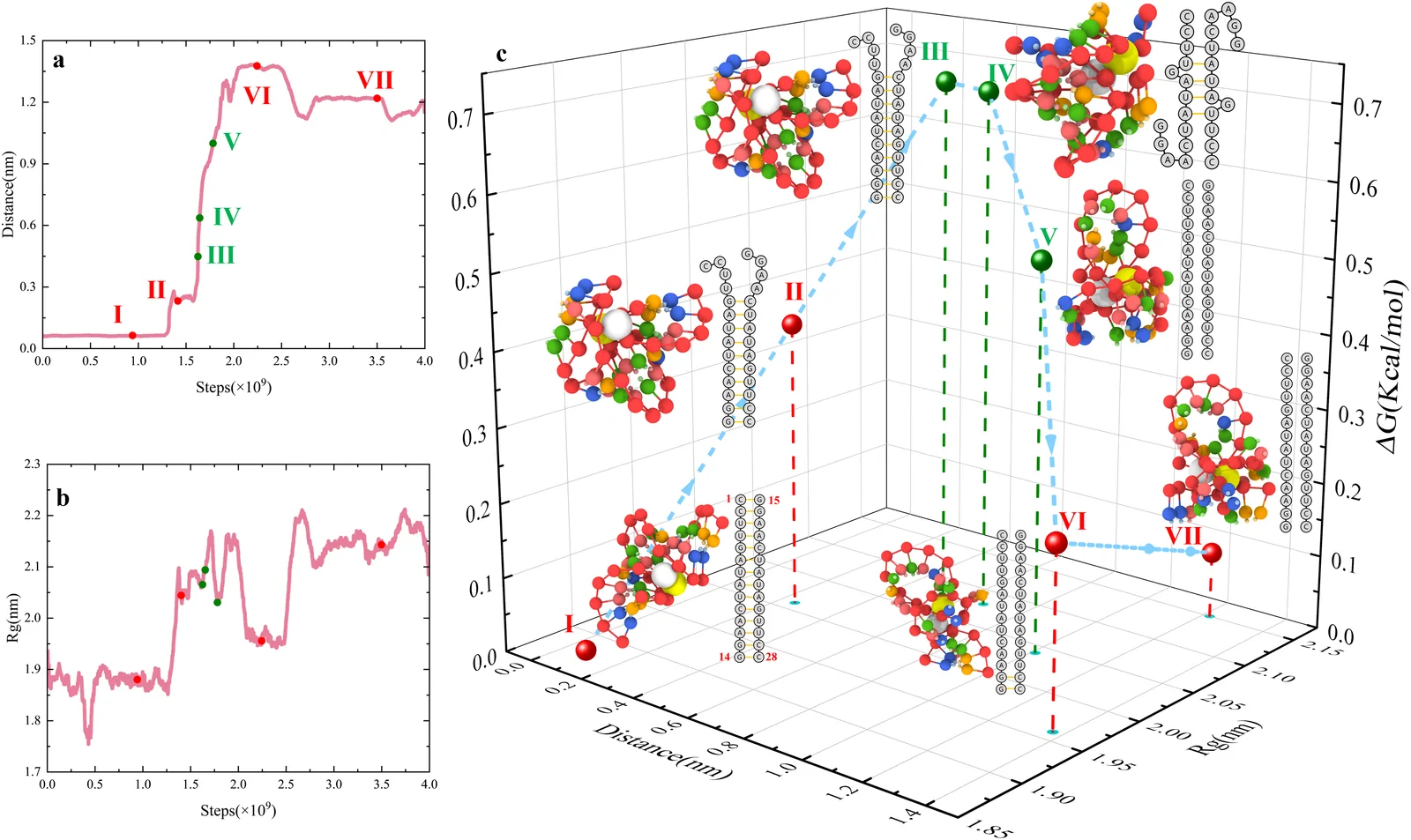
Dynamics of unwinding dsRNA by DDX3X. (a) Temporal evolution of Rg. (b) Temporal evolution of Distance. Sphere color encodes the structural stability of the DDX3X-dsRNA complex: red indicates low-energy, long-lived states, whereas green corresponds to higher-energy, transient states with short-lived lifetime. (c) Representative conformational snapshots along the unwinding trajectory at selected time points. I: 9.39 × 10^8^ step; II: 1.4 × 10^9^ step; III: 1.625 × 10^9^ step; IV:1.6432 × 10^9^ step; V: 1.7824 × 10^9^ step; VI: 2.2441 × 10^9^ step; VII: 3.5 × 10^9^ step. The visualizations integrate three-dimensional complex topology (with the disordered regions of DDX3X omitted for clarity).

In SB state shown in Fig 3c.I, structural visualization of the DDX3X-dsRNA complex demonstrated tight binding of DDX3X to dsRNA, inducing localized bending, and secondary structure of dsRNA remains intact. State Fig 3c.II corresponds to the PU state, characterized by the disruption of 4-bp and bent ssRNA segments that preferentially interact with the D1 and D2 helicase domains, thus exposing the base of the groove regions. The transient state in Fig 3c.III also represents the PU state, but exhibits complex base rearrangement dynamics mediated by DDX3X under thermal fluctuations. In particular, transient disruptions of hydrogen bonding networks and base stacking cause multiple base pair destabilizations, visualized as an unstable configuration. Concomitantly, bending of ssRNA backbone facilitates helicase binding, while the dsRNA adopts hybrid conformations that incorporate both helix and interloop elements.

Progression to the unstable state in Fig 3c.IV was marked by sequential disruption of 3-bp in terminal, with ssRNA adopting circular conformations around D1/D2 domains. This structural rearrangement preserved the four central base pairs in helical configuration, maintaining only short helix elements in secondary structure. The FU state in Fig 3c.V showed complete helical disintegration, with circularized ssRNA strands encapsulating D1/D2 domains and fully exposed bases. The rapid III → IV → V evolutions correlated with abrupt increases in Distance during unwinding (Fig 3a), indicating a successful unwinding event. Post-unwinding relaxation enabled structural reorganization into FU conformation in Fig 3c.VII. Subsequent trajectory revealed reformation of PR state in Fig 3c.VI through terminal RNA base stacking and hydrogen bonding, followed by reversion to FU state in Fig 3c.VII. The interconversion between FU and PR states is maintained by continuous enthalpy-entropy compensation arising from thermal fluctuation. Successful attainment of the FU state requires passing through the transient intermediates (Fig 3c.III and IV), which correspond to unstable, high-energy strand-displacement states.

By comparing successful and unsuccessful unwinding events, we found that successful transition from the SB state to the PU state requires capturing stochastic nucleobase fluctuations at either dsRNA terminus, a process that is independent of DDX3X binding sites. The transition from the PU state to the FU state can proceed via two distinct pathways (Fig C in S1 Appendix): (i) In the SB state, sequential opening of 3–4 base pairs forms the PU state (Fig 3c.II), followed by continuous base pair disruption (maintaining 5–8 opened pairs). (ii) The PU state (Fig 3c.II) undergoes base-rearrangement to form another PU state (Fig 3c.III) and induces rupture of terminal base pairs (Fig 3c.IV), ultimately resulting in complete dsRNA unwinding. This bifurcation mechanism underlies the stochastic nature of DDX3X-mediated dsRNA unwinding, where pathway selection depends on real-time capturing of RNA nucleobase fluctuations.

Notably, the free energy of different states (ΔG; see Materials and Methods) is calculated, and the energy barrier is constructed in a two-dimensional reaction coordinates space defined by *Distance* and *Rg* (Fig 3c). This behavior indicates that the underlying physical mechanism of the unwinding process shares key features with a nucleation-like transition. Successful unwinding corresponds to trajectories that cross this free-energy barrier, whereas trajectories that fail to overcome the barrier instead relax into PU or SB states. This two-dimensional representation highlights that unwinding is not governed by a single structural variable, but instead arises from the coupled evolution of strand separation and global conformational rearrangement. These results implies that thermal fluctuations and entropy gain can provide a driving force for dsRNA remodeling, enabling stochastic barrier crossing.

### Development and evaluation of entropy-UNet

Artificial Intelligence (AI) has emerged as a powerful tool for extracting features from high-dimensional scientific data. In non-equilibrium dynamics, configurational entropy derives from a joint probability distribution over strongly correlated degrees of freedom. Although CRE can be computed deterministically for individual snapshots, estimating the underlying probability distribution, and elucidating how hydrogen bonding, base stacking, and backbone fluctuations collectively shape the entropic landscape, remains impeded by the non-factorizability of these coupled coordinates. This raises the question of whether AI-based approach can learn entropy-related features from such non-equilibrium trajectories. To explore this, we develop Entropy-UNet, a deep-learning framework designed to learn the conformational distribution of dsRNA directly from simulation trajectories, enabling entropy estimation and thermodynamic characterization.

Entropy-UNet (Fig 4a, 4b) was developed to leverage coarse-grained trajectories generated by Metadynamics [32] (see Metadynamics Implementation in S1 Appendix) for constructing physically informed entropy fingerprints. This approach enables precise mapping between transient conformational states and entropy variations. Metadynamics served as the primary sampling strategy, employing predefined CVs to effectively capture conformational states for deep learning applications. We generated two distinct datasets using Rg^system^ and Dist as CVs, yielding 40,000 and 30,000 conformational samples, respectively. These datasets were systematically partitioned into training, validation, and test subsets at an 8:1:1 ratio. The model underwent 200 training epochs with a learning rate of 0.001, demonstrating robust performance on test data (KLD = 0.0887; CE = 3.8189; BS = 0.0014; CS = 0.9727).

**Fig 4:**
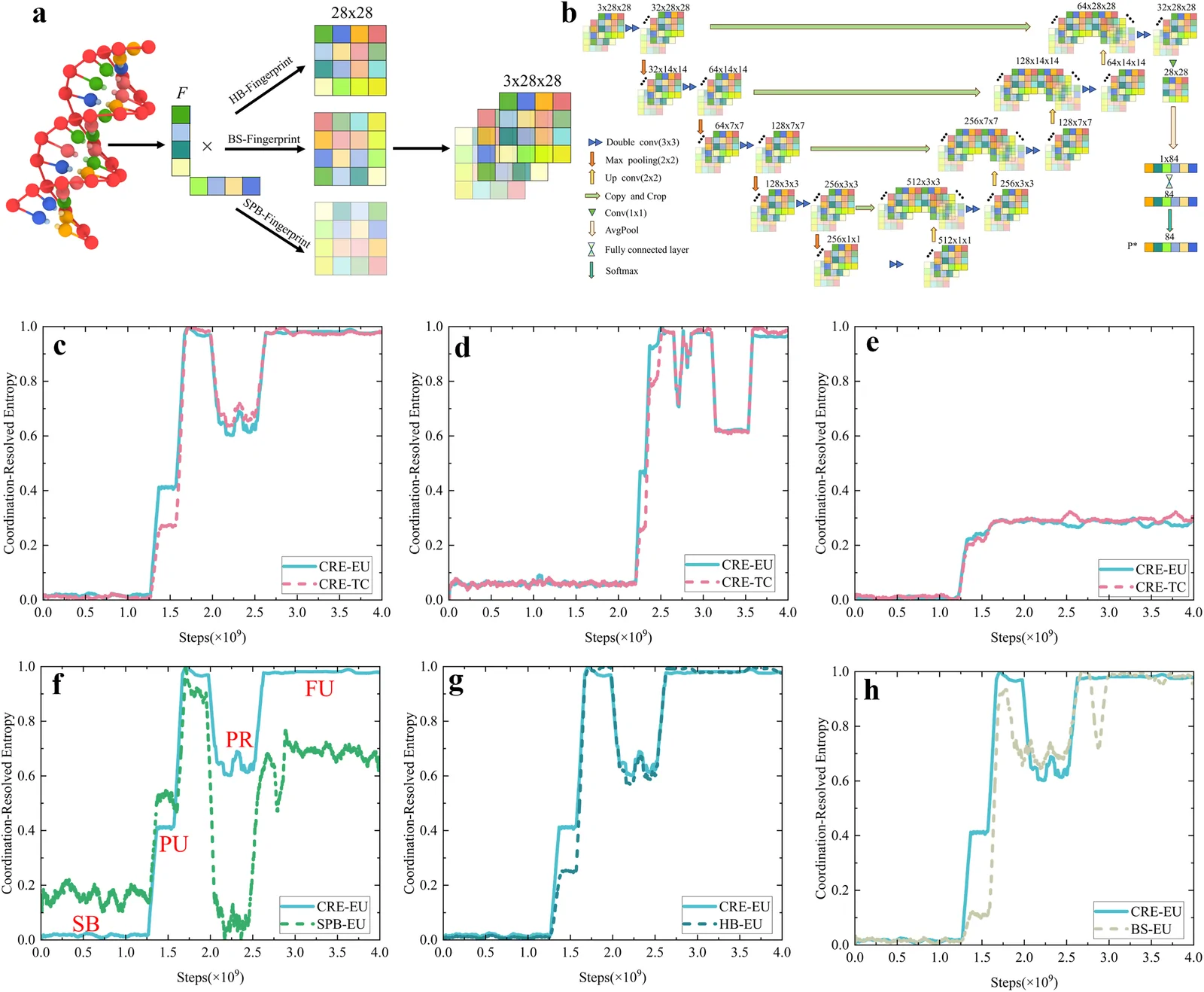
Development and evaluation of Entropy-UNet. (a) Schematic of entropy fingerprints. (b) Entropy-UNet Architecture Overview. (c)~(e) Comparative Analysis of CRE-EU and CRE-TC across three independent test datasets. (f)~(g) Entropic contributions of SPB, HB, and BS were Entropy-UNet resolved.

For rigorous validation, we evaluated three independent test cases. Two diverse successful unwinding trajectories (Fig 4c, 4d) with significant conformational transitions, and an unsuccessful trajectory (Fig 4e) persistently occupying the PU state. Quantitative comparison between CRE-EU (CRE predicted from Entropy-UNet) with CRE-TC (CRE-Theoretical Calculations) curve revealed strong predictive accuracy. Fig 4c illustrates the complete SB → PU → FU → PR → FU transition pathway during dsRNA unwinding by DDX3X. Entropy-UNet precisely tracked entropy changes across states, with minor overestimations observed for PU states in Fig 4c and 4d. Notably, predictions for PU states in test cases Fig 4e showed excellent agreement with theoretical CRE values.

To evaluate whether Entropy-UNet captures meaningful physical interactions, we analyzed its architecture to assess how Hydrogen Bonding (HB), Base Stacking (BS), and Sugar-Phosphate Bead (SPB) fingerprints influence PU state predictions. The encoder’s final bottleneck layer compresses spatial fingerprints into high-level semantic representations, while the decoder up-samples with residual skip-connections. Guided by this, we applied Grad-CAM [33] to the penultimate encoder layer to examine entropy fingerprint activation. Three representative PU state instances were analyzed (Fig D in S1 Appendix), revealing that SPB features exhibit higher semantic sparsity than HB and BS. Incomplete SPB representations correlated with prediction errors, causing over- or underestimation of entropy in PU and PR states (Fig 4c). These findings indicate that Entropy-UNet primarily learns physically meaningful interactions, including BS, HB, and backbone electrostatics, while effectively filtering non-essential background features.

Overall, Entropy-UNet demonstrated consistent predictive reliability across all test conditions, establishing a novel framework linking conformation with entropy. Notably, in Fig 4d, the model captured complex transition dynamics, including extended SB occupancy followed by rapid PU → FU → PR → FU conversion, highlighting its structural resolution capability. Using “soft pixel” feature images, we compared Entropy-UNet with a baseline CNN (without residual connections) and a Transformer-Encoder [34] on an independent test set (FigE in S1 Appendix). While overall performance was comparable to the CNN, Entropy-UNet excels at capturing biophysically meaningful interactions, extending advances in structure-aware deep learning exemplified by UFold [35]. Although the Transformer-Encoder better identified PU states, it underperformed for PR states. In contrast, Entropy-UNet maintains robust, balanced performance across conformational states, providing consistent interpretability and reliability for structural bioinformatics applications.

### Distinct contributions of CRE predicted by Entropy-UNet

The CRE metric employs coordination numbers of dsRNA beads as spatially resolved indicators of local structural order, quantifying entropy through cooperative interactions among proximal particles. This approach inherently captures the coupled thermodynamic contributions of hydrogen bonding, base stacking, and sugar-phosphate backbone electrostatic interactions. Entropy-UNet employs entropy fingerprints explicit spatial adjacency modeling between bead types to dynamically resolve their distinct spatial information. To disentangle these effects, we systematically evaluated their individual contributions to CRE by computing independent CRE values for each component. Our analysis reveals three key findings (Fig 4f-4h): First, SPB-EU successfully distinguished SB and PU states, albeit with systematic overestimation, while detecting PR and FU states with consistent underestimation based on entropy profile analysis; Second, HB-EU demonstrated exceptional agreement with reference values, accurately identifying all four states (SB, PU, PR, FU) with only minor PR-state underestimation; Third, BS-EU showed intermediate performance, resolving all states with moderate accuracy (superior to SPB-EU but inferior to HB-EU) while slightly underestimating PU-state entropy. Leveraging Entropy-UNet’s precise, interaction-specific entropy decomposition, we established the thermodynamic hierarchy of contributions: hydrogen bonding (primary)> base stacking (secondary)> backbone electrostatics (tertiary), with cooperative HB and BS interactions dominating the overall entropy landscape.

## Discussion and conclusion

In this study, our integrated computational approach, combining the coarse-grained HTri-CG RNA model with Entropy-UNet, reveals that DDX3X-mediated dsRNA unwinding in the absence of ATP occurs via a stochastic, entropy-driven process. The HTri-CG RNA model reveals that the unwinding dynamics is primarily governed by DDX3X-induced disruption of Watson-Crick base pairing and base stacking interactions through thermal fluctuations, which facilitates relative motion between the two ssRNA strands and promotes structural melting by overcoming high-energy intermediate states. Entropy-UNet’s high-resolution decomposition further shows that hydrogen bonding and base stacking interactions are the primary determinants affecting RNA conformational reorganization during DDX3X-mediated dsRNA unwinding. Remarkably, this mechanism mirrors well-characterized DNA melting processes, such as those mediated by bacteriophage T4 gene 32-protein [36], highlighting its generalizability to RNA systems. Meanwhile, this unwinding mechanism aligns with the theoretical framework of passive unwinding of helicases proposed by Thirumalai et al. [37,38]. It further reveals a significant evolutionary strategy: for helicases lacking powerful active disruption capabilities, utilizing environmental thermal fluctuations for unwinding represents an efficient and conserved physically economical solution.

A key prediction of the proposed entropy-driven mechanism is that DDX3X can promote rare, transient strand-displaced intermediates even in the absence of ATP. This prediction can be tested on short, low-stability dsRNA substrates under ATP-free conditions, with particular attention to detecting transient PU intermediates and their occurrence probabilities. Several relevant experimental approaches have already been applied to this system. Dual-color fluorescence cross-correlation spectroscopy (dcFCCS), single-molecule FRET and fluorescence anisotropy, and mass photometry have resolved DDX3X RNA-protein cluster formation, RNA capture, and strand release in vitro [12]. Complementing these approaches, site-specific ^19^F NMR ribose probes can report on local and global dsRNA-ssRNA interconversion with minimal interference from protein background; previous studies using such probes have shown that DDX3X can bind a locally unwound duplex and adopt an ssRNA-bound closed conformation [10]. Together, these complementary approaches provide a promising route to directly test key predictions of the stochastic, entropy-driven unwinding mechanism proposed here.

While our integrated approach successfully reproduced experimental observations of DDX3X-mediated dsRNA unwinding, several methodological disadvantages and advantages should be acknowledged:

(i). HTri-CG RNA Model:

Compared to the TiRNA [39], SimRNA [40], Vfold [41], IsRNA [42] and oxRNA models, the HTri-CG model sacrifices some structural precision, including the accurate description of base stacking and hydrogen bond anisotropy. This trade‑off originates from its three‑bead coarse‑graining strategy, which prioritizes computational efficiency for probing dynamic molecular mechanisms of RNA‑protein interactions, rather than aiming for de‑novo RNA 3D‑structure prediction. Nevertheless, its reliability has been validated across multiple RNA systems, including a short helical S1S2(28nt) dsRNA, a long helical XY (73nt) dsRNA with two interloop elements, and a 5-bp stem tetraloop hairpin. Although the model does not explicitly represent non-canonical base pairs, this omission is justified empirically for short dsRNA, given that 70–80% of base pairs in the PDB are WC, with the remaining fraction forms non‑canonical base pairs [43]. Notably, the isotropic Lennard-Jones potential used in our coarse-grained model to describe both stacking and hydrogen-bonding interactions tends to produce overly ordered intra-strand stacking conformations. Despite this simplification, quantitative benchmarking against all-atom simulations demonstrates that our model remains applicable to dsRNA. For instance, the averaged end-to-end distance of a 23-bp dsRNA predicted by our model differs from the all-atom results by approximately 15% (Fig F in S1 Appendix). Taken together, the HTri-CG model offers an effective balance between accuracy and efficiency for simulating dsRNA unwinding dynamics.

(ii). Modeling Protein-RNA Interactions:

According to Song and Ji [7], interactions between the D1/D2 domains and nucleic acids are the primary determinants of DDX3X-mediated unwinding. This insight enabled the successful simulation of dsRNA unwinding by DDX3X. The coarse-grained model of DDX3X was constructed using the sticker-and-spacer framework (Fig G in S1 Appendix), a simplified representation known for its scalability. When extending this DDX3X-RNA framework to other protein–RNA complexes—such as the helicases DDX5 [44] and eIF4A [45]—precise definition of the interaction sites and binding modes between the helicase and nucleic acid becomes essential. Consequently, the approach depends heavily on the availability of high-resolution crystal structures of such complexes. Recent advances in predictive accuracy for RNA–protein interactions, achieved through algorithms like AlphaFold3 [46], may help mitigate this limitation.

(iii). Entropy-UNet Performance:

Entropy-UNet utilizes HB, BS, and SPB entropy fingerprints to effectively characterize the three primary RNA interaction types, enabling both accurate entropy prediction and entropy contributions decoupling (Hydrogen bonding > Base stacking > Backbone electrostatics). However, while the architecture maintains translation invariance and hierarchical feature learning capabilities, it lacks explicit rotational symmetry, potentially limiting its robustness when analyzing PU conformational ensembles. Furthermore, the dimensionality of entropy fingerprints is inherently constrained by the bead count in the coarse-grained dsRNA representation.

In our future work, we will continue to delve into RNA‑protein complex systems and persistently expand our model capabilities. Within the current three‑bead coarse‑grained framework, we will explore the potential for advancing general RNA 3D‑structure prediction and reliable structural‑stability evaluation, which will require additional structural restraints, refined parameters for base stacking and hydrogen‑bond interactions, and expanded validation datasets covering diverse RNA systems. In addition, we will also address open questions such as RNA systems extensively featuring non‑canonical structures or more complex protein condensate systems.

## Materials and methods

### Free energy and coordination-resolved entropy

To characterize the free energy of DDX3X-dsRNA unwinding in MD simulations, we performed binning-based statistical analysis using two essential geometric parameters extracted from the MD trajectories: (1) the radius of gyration of the entire system ($Rg=\sqrt{\frac{\text{1}}{N}\sum_{i=\text{1}}^{N}|r_{i}-R_{com}{|}^{\text{2}}}$, *R*_*com*_ is center of mass coordinate vector) and (2) the center-of-mass distance between two strands of the dsRNA ($Distance=|R_{com}^{X}-R_{com}^{Y}|$). This Visited States Method [47] directly utilized the time-evolution of these MD-calculated structural metrics to construct the free energy. While this method for obtaining complete free energy generally requires exhaustive sampling of high-barrier regions, our extended simulations (4 × 10^9^ steps) successfully captured key metastable intermediates in DDX3X-dsRNA unwinding, ensuring robust free energy reconstruction. *Rg* and *Distance* dimensions were discretized into 100 equally spaced bins per axis, generating a 10,000-bin grids. The discrete joint probability distribution *P (Rg*_*i*_*, Distance*_*j*_*)* for bin (*i,j*) was calculated as:


$$\begin{array}{c} \begin{array}{c} \;P\left(Rg_{i},\;Dis{tance}_{j}\right)=\frac{N_{ij}}{N_{total}} \end{array} \end{array}$$ (1)

where *N*_*ij*_ denotes the number of MD frames falling into the bin (*i*, *j*), and *N*_*total*_ = 4.0 × 10^4^ corresponds to the total number of frames sampled from MD trajectories. Free energy was subsequently derived via:


$$\begin{array}{c} \begin{array}{c} \Delta G=-\text{0}.\text{0}\text{0}\text{1}N_{A}k_{B}Tln\frac{P\;\left(Rg_{i},\;Dis{tance}_{j}\right)\;}{\;{P(Rg,\;Distance)}_{max}} \end{array} \end{array}$$ (2)

with *T* = 300K, *N*_*A*_ is the Avogadro constant and *P (Rg, Distance)*_*max*_ as the maximum probability bin to ensure that the lowest free energy *ΔG* = 0 kJ/mol. The factor 0.001*N*_*A*_ serves as a unit‑conversion term. Multiplying this factor with the single-molecule thermal energy *k*_*B*_*T* converts energy from per-molecule units (J) into molar-scale free-energy in kJ/mol. For figure visualization, these raw kJ/mol values are further converted to kcal/mol using the conversion factor 1 kcal/mol = 4.184 kJ/mol.

Our analysis reveals that DDX3X-mediated dsRNA unwinding originates from thermal fluctuations, disrupts the hydrogen-bonding network and base stacking interactions, then facilitating structural melting through preferential binding to the single-stranded denatured. Meanwhile, the conformational changes during chain dissociation processes occur at ultrafast timescales, the subtle structural variations in these conformations remain traceable and inherently contain valuable information-particularly through coordination number alterations-that reflects thermal fluctuations. To quantify these conformational changes, we employ an information entropy framework through the following protocol:

(i) Calculate a single‑bead smoothed version of the radial distribution function by [48]


$$\begin{array}{c} \begin{array}{c} g_{m}^{i}(r)=\frac{\text{1}}{\text{4}\pi \rho r^{\text{2}}}\sum_{j}\frac{\text{1}}{\sqrt{\text{2}\pi \sigma^{\text{2}}}}e^{-\frac{{\left(r-r_{ij}\right)}^{\text{2}}}{\left(\text{2}\sigma^{\text{2}}\right)}} \end{array} \end{array}$$ (3)

where *r*_*ij*_ is the distance between beads *i* and *j*, *j* are the neighbors of bead *i*, and σ is a broadening parameter.

(ii) Determine single-bead coordination numbers via integration of Eq. 4;


$$\begin{array}{c} \begin{array}{c} n_{i}(r)=\text{4}\pi \rho \int_{\text{0}}^{r}g_{m}^{i}(r)r^{\text{2}}dr \end{array} \end{array}$$ (4)

Where *ρ* is localized density of bead, *r* = 2.1nm is cutoff distance.

(iii) Establish coordination number probability of single-bead through Eq. 5;


$$\begin{array}{c} \begin{array}{c} P(i)=\frac{n_{i}(r)}{\sum_{i}^{n}n_{i}(r)} \end{array} \end{array}$$ (5)

(iv) We use Shannon entropy (Eq. 6) followed by normalization to obtain coordination-resolved entropy (CRE).


$$\begin{array}{c} \begin{array}{c} CRE=-\sum_{i}^{n}P(i){\text{log}}_{\text{2}}P(i) \end{array} \end{array}$$ (6)

### Entropy fingerprint

In supervised learning, the dual challenge of feature engineering and label engineering persists, where intrinsic coupling between feature-label pairs necessitates coordinated design strategies. To address this, we developed an entropy fingerprint encoding scheme termed “soft pixels” to quantify local order of beads [48], while implementing a “soft labeling” system based on bead coordination number probability distributions in dsRNA. Entropy-UNet architecture employs a deep neural network to perform prediction of coordination-resolved entropy from input entropy fingerprints, establishing an entropic mapping between local order landscapes and coordination patterns of beads. The entropy fingerprint is obtained by


$$\begin{array}{c} \begin{array}{c} F_{i}(r)=-\sum_{i}^{n}\text{2}\pi \rho k_{B}\int_{\text{0}}^{r_{m}}\left[g_{m}^{i}(r)\text{ln}g_{m}^{i}(r)-g_{m}^{i}(r)+\text{1}\right]r^{\text{2}}dr \end{array} \end{array}$$ (7)

Where *ρ* is localized density of system, r = 3.5 nm is cutoff distance, and *k*_*B*_ is a Boltzmann constant. Fig 4a demonstrates the computational pipeline for constructing entropy fingerprint representations. Specifically, the entropy fingerprint F of HB, BS, and SPB are initially encoded as one-dimensional vectors (length = 28). Each vector undergoes tensor product transformation through Kronecker self-multiplication *F* ⊗ *F*^*T*^, generating 28 × 28 matrices that capture second-order entropy correlations, then matrices is normalized to [-1,1]. These matrices are subsequently stacked along the channel dimension to form a three-dimensional (3, 28, 28) tensor representation, termed “soft pixel images”, where each channel corresponds to distinct bead-type entropy landscapes. Entropy-UNet architecture’s novel input representation demonstrates three principal advantages for coordination-resolved entropy prediction:

(i). Spatially resolved interaction modeling: The tensor-based “soft pixel” representation explicitly encodes three fundamental interaction types - base stacking, base pairing, and electrostatic interactions - through distinct feature channels.
(ii). Cross-scale correlation emergence: The Kronecker product operation *F* ⊗ *F*^*T*^, inherently induces non-local interaction patterns from local entropy fingerprints. This mathematical transformation converts 1D bead-specific entropy vectors into 2D pairwise correlation matrices, where the off-diagonal elements *(i, j)* explicitly quantify entropic coupling between spatially separated beads *i* and *j*, thereby establishing spontaneous long-range connectivity beyond the original local descriptors.
(iii). Architectural compatibility: The grid-structured input enables implementation of fully convolutional neural networks, providing inherent advantages in processing high-dimensional conformational data through translational equivariance and hierarchical feature learning.

Other methods are described in S1 Appendix, including The Coarse-Grained Modelling, Replica exchange molecular dynamics Implementation, Metadynamics Implementation, dsRNA and Hairpin constructs and Entropy-UNet network architecture.

## Supporting information

S1 Appendix Supplementary information for “ATP-independent entropy-driven dsRNA unwinding by DDX3X revealed by coarse-grained simulations and deep learning”. (DOCX)
