# Supplementary material for: ATP-independent entropy-driven dsRNA unwinding by DDX3X revealed by coarse-grained simulations and deep learning
Source: PLoS Comput Biol. 2026 Oct 30;22(10):e1014928. doi: 10.1371/journal.pcbi.1014928 (PMC13734570; doi:10.1371/journal.pcbi.1014928)
Supplement: Supplementary file 1 [file pcbi.1014928.s002.pdf]

Reviewers' comments:

Reviewer's Responses to Questions

Comments to the Authors:

Please note here if the review is uploaded as an attachment.

Reviewer #1: Overview:

Wang et al. investigate the physical mechanism behind the ATP independent double-stranded RNA unwinding by the DEAD-box helicase DDX3X. The authors introduce a three-bead per nucleotide coarse-grained model (HelixTriad) that incorporates base-stacking, hydrogen bonding, and backbone electrostatics that is similar to those previously developed in the Thirumalai group over the past 20+ years. Using a combination of Langevin dynamics and metadynamics, they observe that DDX3X-mediated unwinding is an inherently stochastic, entropy-driven process that overcomes the net positive enthalpy barrier through thermal fluctuations and conformational entropy gain. The authors also develop a U-Net-based deep learning architecture (Entropy-UNet) to predict coordination-resolved entropy (CRE) from “soft pixel fingerprints. Using the feature-attribution analysis, they rank thermodynamic factors driving unwinding: hydrogen bonding > base stacking > backbone electrostatics.

The manuscript is generally well-organized and combines classical statistical mechanics with modern deep learning architecture to address an intriguing biophysical question. However, there are several methodological and analytical issues that must be addressed prior to publication:

Major Comments:

1.The authors state that their HelixTriad coarse-grained model is an extension of previous three-bead-per-nucleotide frameworks proposed in previous studies by explicitly incorporating hydrogen bonding and base-stacking interactions. In fact, base stacking interactions were incorporated in the very first such model in Hyeon and Thirumalai PNAS 2005 and hydrogen bonding was explicitly incorporated later in Denesyuk and Thirumalai, JPCB 2013.

Reply: Thank you for pointing out this important historical context. In the three-bead RNA model [PNAS, 102 (19) 6789-6794], explicit base-stacking potential was included, whereas base-pairing stabilization was achieved implicitly via native-contact interactions without a dedicated hydrogen-bonding term. In the revised TIS model [J. Phys. Chem. B (2013) 117 (17): 4901–4911], an explicit directional hydrogen-bonding potential was added on the base bead while retaining base-stacking. However, since both interactions act on the same base bead, they still cannot be physically decoupled.

In our HelixTriad coarse-grained model, we decoupled base-stacking and hydrogen-bonding interactions by adopting the three-bead coarse-graining strategy for DNA from the Mittal group [JCTC 20,1717-1731 (2024)]. Specifically, the phosphate and ribose moieties are coarse-grained into one combined phosphate-sugar bead, the nucleobase is represented by a base bead, and an additional virtual hydrogen-bond bead is introduced. Base-stacking and base-pairing

hydrogen-bonding interactions are therefore explicitly separated onto distinct beads. We wish to clarify that hydrogen-bonding and base-stacking themselves are not newly proposed interactions in the revised manuscript; rather, the key advance lies in their spatial separation within the model.

Furthermore, the architecture of our model exhibits favorable expandability. For example, electrostatic interactions, hydrogen bonds, and salt bridges between DDX3X protein and RNA mainly occur at phosphate and ribose groups. Our bead-mapping scheme facilitates future coarse-grained modeling of protein-RNA complexes.

The manuscript is revised as follows:
Page 2, Lines 38-41: To bridge this gap, we developed HelixTriad coarse-grained RNA model by adopting the three-bead-per-nucleotide framework proposed in previous studies, where base-stacking and hydrogen-bonding interactions are physically decoupled via distinct base and hydrogen-bonding beads.

2.The authors note in Discussion and Conclusion section that non-canonical base pairs are not explicitly represented and that this omission is justified empirically but give no reference or further explanation. There really needs to be a much more fuller reasoning and description of what they did. Did non-canonical base pairs really have no hydrogen bonding or base stacking interactions in their simulations? How many non-canonical base pairs are in DDX3X? Local RNA strand displacement often involves non-canonical intermediate states or transient bulge configurations, which would impact their results of partially wound (PU) states.

Reply: Thank you for your question. According to recent literature [Nature Chemistry, 14, 775-785 (2022)], approximately 70-80% of base pairs in the PDB are Watson-Crick pairs, and the rest are non-canonical base pairs. Therefore, the effects of non-canonical base pairs on long or densely packed dsRNA systems can’t be ignored.

Recent studies showed that DDX3X can unwind dsRNA substrates up to 23-bp. It exhibits no structural selectivity toward canonical ssRNA, dsRNA or hairpin RNA, with specific recognition largely restricted to G-quadruplex motifs [Nature Communications, 16, 7762 (2025); Nature Communications, 10, 3085 (2019)]. Available structural data for DDX3X-RNA complexes are too limited to enable meaningful quantitative statistics on non-canonical base pairs, largely because these complexes typically involve short dsRNA constructs.

In our work, we investigated the melting or unwinding of short dsRNA. The dsRNA initially adopts a perfectly paired conformation with canonical A-U and C-G base pairs. During the melting or unwinding process, however, non-canonical bases may transiently form. In our simulation, base-stacking interactions involving non-canonical bases are fully preserved, while only hydrogen-bonding pairing terms for such bases are disabled. This treatment is motivated by the physical consideration that, for the short dsRNA, inter-strand interactions are expected to diminish rapidly as the double helix dissociates, whereas intra-strand interactions can be preserved. Admittedly, it is difficult to accurately determine how many non-canonical bases arise during the dynamical process.

Notably, our results also reveal the existence of local RNA strand displacement and transient bulge configurations, which at least partially validates our treatment of non-canonical bases. Nevertheless, a more precise description of non-canonical base pairs will be necessary for long dsRNA systems.

The manuscript is revised as follows:

SI Page 1, Lines 32-37: In our simulation, base-stacking interactions involving non-canonical bases are fully preserved, while only hydrogen-bonding pairing terms for such bases are disabled. This treatment is motivated by the physical consideration that, for the short dsRNA, inter-strand interactions are expected to diminish rapidly as the double helix dissociates, whereas intra-strand interactions can be preserved.

Page 14, Lines 23-25: Although the model does not explicitly represent non-canonical base pairs, this omission is justified empirically for short dsRNA, given that 70–80% of base pairs in the PDB are WC, with the remaining fraction forms non-canonical base pairs.

3.A U-Net architecture is traditionally designed for semantic segmentation of 2D grid image data. The authors demonstrated that Entropy-Unet performs well, as shown in Fig. S5, there appears to be very subtle differences compared to other models. Also, as mentioned in the discussion, 2D convolutions are invariant to translation but biomolecules also require rotational invariance. The authors should describe how they addressed it to prevent artifactual bias.

Reply: Thank you for your question. We did not specially address or implement rotational invariance for our Entropy-UNet model. Importantly, our target DDX3X-dsRNA complex is an asymmetric biomolecular system without rotational symmetry. There exists a well-defined native binding pose between protein and dsRNA; different rotational orientations of the whole complex correspond to physically distinct asymmetric configurations. Therefore, the absence of rotational invariance of 2D convolutions would not introduce severe artifactual bias for the present task.

4.The authors report the previously unrecognized physical mechanism for RNA duplex unwinding. Can the authors also suggest experiments that can check their predictions?

Reply: Thank you for your question. DDX3X mediates dsRNA unwinding through local perturbation and is known to form functional nanometer-scale RNA-protein clusters (RPCs) even without ATP [Current Biology, 34, 5714-5727.e5716 (2024)]. For experimental validation of our simulation predictions, we suggest testing short, low-stability dsRNA substrates under ATP-free conditions, where cooperative assembly of DDX3X into nanoscale RPCs is expected. Single-molecule tools including dcFCCS and smFRET-anisotropy can detect the transient PU intermediates predicted in our work, while mass photometry or DLS can characterize RPC formation and sizes.

Minor Comments:

1) In abstract (line 28), "dominate factor" should be "dominant factor".

Reply: **Page 1, Line 28:** dominate factor→ dominant factor

2) The box sizes should be reported as 30 x 30 x 30 nm3, not 303 nm3.

Reply: **Page 4, Line 18 and Page 5, Line 35:** 30^3 nm^3→ 30 x 30 x 30 nm^3

3) In the funding section, it should read "Ministry of Education of China", not "Chain".

Reply: **Page 20, Line 25**: Chain → China

4) In Fig. 1d caption, NaCL should be NaCl.

Reply: **Page 3, Line 26**: NaCL→ NaCl

Reviewer #2: For “ATP-Independent Entropy-Driven dsRNA Unwinding by DDX3X Revealed by Coarse-Grained Simulations and Deep Learning” by Wang et al.

The authors have developed a physics-based coarse-grained (CG) RNA model with incorporating weak and specific interactions between DEAD-box RNA helicases (DDX3X) and dsRNA, to explore the inherently stochastic nature of dsRNA unwinding. The work revealed that the dsRNA unwinding process need to overcome an energy barrier, whereas thermal fluctuations and entropy gain provide a driving force for RNA remodeling. Moreover, the work revealed that dsRNA separation proceeds through rare but obligatory strand-displacing intermediates by DDX3X. The work provides a deep understanding on how DDX3X promotes dsRNA unwinding. The manuscript is well-organized. I am pleased to recommend its acceptance on PlosCB after the authors properly address the following issues.

1, The authors proposed a three-bead CG model for dsRNA and validating the model through dsRNA thermal stability and elasticity. The three-bead representation in the model is different from those in existing CG models for RNAs such as SimRNA, Vfold, IsRNA, TiRNA and etc. It would be rather valuable if the authors could add sentences to state the rational for such CG representation and to discuss the possibility to extending the present model to a more complete model for predicting RNA 3D structures or predicting RNA structure stability.

Reply: Thank you for your question. Compared with existing RNA coarse grained models including SimRNA, Vfold, IsRNA, and TiRNA, our model represents each nucleotide with three beads: a phosphate sugar bead, a base bead, and a hydrogen bond bead. This setup physically decouples electrostatic interactions, base stacking interactions and hydrogen bond interactions, yielding a higher coarse graining level. Our current coarse-grained model is tailored for short dsRNA systems and is suitable for investigating duplex unwinding dynamics, which also is suitable to be extended to protein-RNA complex systems.

There is potential for future extension within the current three bead framework, while considerable challenges remain. The present parameters are mainly fitted for short dsRNAs. To achieve general RNA 3D structure prediction and reliable evaluation of RNA structural stability, additional structural restraints, refined parameters for base stacking and hydrogen bond interactions, as well as enlarged validation datasets covering diverse RNA systems will be required. Such comprehensive extension is beyond the scope of the current work and will be considered in our future studies.

Correspondingly, we have added relevant discussions on the three bead HTri CG model in its subsection, as well as model extension outlooks in the Future Work section.

The manuscript is revised as follows:
Page 14, Lines 15-20: Compared to the TiRNA, SimRNA, Vfold, IsRNA and oxRNA models, the HTri-CG model sacrifices some structural precision, including the accurate description of base stacking and hydrogen bond anisotropy. This trade-off originates from its three-bead coarse-graining strategy, which prioritizes computational efficiency for probing dynamic molecular mechanisms of RNA-protein interactions, rather than aiming for de-novo RNA 3D-structure prediction.

Page 15, Lines 8-15: In our future work, we will continue to delve into RNA-protein complex systems and persistently expand our model capabilities. Within the current three-bead coarse-grained framework, we will explore the potential for advancing general RNA 3D-structure prediction and reliable structural-stability evaluation, which will require additional structural restraints, refined parameters for base stacking and hydrogen-bond interactions, and expanded validation datasets covering diverse RNA systems. In addition, we will also address open questions such as RNA systems extensively featuring non-canonical structures or more complex protein condensate systems.

2, I still suggest that the authors can add a brief description for the proposed structure model in the main text although the model is described in details in the Supporting Information. It would be very helpful for PlosCB readers.

Reply: Thank you for the suggestion. We have added a brief description of the three-bead HTri-CG model architecture in the Development and Validation of the HTri–CG Model section. Full model parameters are still provided in the Supporting Information **(S2-S5 Tables)**.

The manuscript is revised as follows:
Page 4, Lines 4-12: In this representation, each nucleotide is decomposed into three physically distinct beads: a sugar-phosphate backbone bead responsible for backbone electrostatics, a base bead for base-stacking interactions, and a dedicated hydrogen-bond bead that mediates Watson-Crick (WC) base-pairing. This three-bead design enables physical decoupling of backbone electrostatic, base-stacking, and hydrogen-bonding interactions, rather than lumping multiple chemical contributions into a single bead. All bead types, interaction potentials, and full parameter sets are comprehensively documented in the Coarse–Grained Modelling of Supplementary Information (SI).

3, In SI, the authors described the energy components of their model in details, and there are some parameters for the energy components. The author may need to add a table in the SI to show the values for the parameters, which would be helpful for potential users.

Reply: We appreciate this useful suggestion for future users of our model. Complete parameter values for all energy components of the HTri-CG model have already been summarized in S2-S5 Tables within the Supporting Information, including base-stacking, hydrogen-bonding, sticker, bonded-bond and bonded-angle parameters. To improve readability and help potential users rapidly locate these tables, we have added explicit cross-references to S2-S5 Tables in the main text of the "The Coarse-Grained Modelling" subsection of SI, to explicitly point readers to these parameter tables.

4, The authors proposed an Entropy-UNet method to predict coordination-resolved entropy (CRE) to capture the conformational changes associated with dsRNA unwinding. I am not sure whether the method can be extended or directly applicable for RNA conformation folding. It would be valuable for readers if the authors can clarify the issue, given that the code of Entropy-UNet has been

available at github.

Reply: The present Entropy-UNet implementation is tailored for dsRNA unwinding under the HTri-CG framework and cannot be directly applied to general RNA-folding tasks out-of-the-box. While the underlying principle of learning structural probability distributions is conceptually generalizable to other macromolecules, our existing code is constrained by system-specific inputs such as the RNA chain length and HTri-CG entropy fingerprint format. Successful adaptation to new systems requires new training data, revised fingerprints, input-dimension adjustment and hyper-parameter retuning. We have provided the code of Enropy-UNet and corresponding usage warnings on our GitHub repository (https://github.com/BeastyWK/Entropy-UNet).

5, some very minor issues:
A, In the caption of Fig. 1, the related experimental references may need to be added;

Reply: We have added the experimental references (20-22) in the caption of Fig 1.

B, In Equation 2, what is the unit of \Delta G? It would be helpful if the authors could explain the coefficient before kBT such as 0.001N_A;

Reply: In Equation (2), the calculated ΔG is reported in kJ/mol. The term k_BT represents the characteristic thermal energy scale per molecule, with units of J. The factor N_A converts per-molecule energy into molar energy with units J·mol−1. Multiplying by 0.001 further transforms J/mol into kJ/mol. Collectively, the product $0.001N_A\ k_BT$ yields molar free-energy in kJ/mol. For visualization in Figure 3c, these raw kJ/mol outputs were post-processed and converted into kcal/mol using 1 kcal/mol=4.184 kJ/mol.

We have added this unit explanation and physical interpretation of the prefactors immediately following Equation (2) in the manuscript.

The manuscript is revised as follows:
Pages 15-16, Lines 39-42;1: The factor $0.001N_A$ serves as a unit-conversion term. Multiplying this factor with the single molecule thermal energy k_BT converts energy from per molecule units (J) into molar scale free energy in kJ/mol. For figure visualization, these raw kJ/mol values are further converted to kcal/mol using the conversion factor 1 kcal/mol=4.184 kJ/mol.

C, A minor inconsistency exists between the main text and supporting information on the corresponding authors.

Reply: We have corrected this inconsistency.

Reviewer #3: In this manuscript, Wang et al. investigate ATP-independent unwinding of a double-stranded RNA by RNA helicase DDX3X using a new coarse-grained model. The authors validated the model against melting curves of two dsRNAs and then applied it to simulations of DDX3X–dsRNA complex. From simulations, they identify several conformational states and argue that dsRNA unwinding occurs stochastically through thermally driven fluctuations and is primarily entropy-driven. To support this, the authors introduce a coordination-resolved entropy (CRE), defined from the distribution of local coordination numbers, and use this quantity to monitor structural disorder during unwinding. They further develop a deep-learning model, Entropy-UNet, to predict CRE and to decompose the apparent contributions of hydrogen bonding, base stacking, and backbone interactions.

The topic is potentially interesting, and the combination of coarse-grained simulations with ML-based structural analysis could in principle provide useful insight into RNA-protein interactions.

I previously reviewed this manuscript for another journal. Having read it again, I am pleased to see that the authors have revised it in line with the suggestions made by the reviewers, including myself. However, there are still several issues that remain unresolved.

Reply: We sincerely thank the reviewer for taking the time to re-evaluate our manuscript and for acknowledging the revisions we have made. We greatly appreciate the continued constructive feedback.

Major points:

(1) The first point remains about the model validity. It remains unclear whether the coarse-grained model can represent general dsRNA properties, particularly unwinding dynamics. I appreciate that the authors have added another dsRNA system, so that two dsRNAs in total are now tested for their ability to reproduce melting temperatures. However, to investigate enzyme-mediated unwinding, particularly when decomposing the contributions of different energetic components such as stacking, hydrogen bonding, and backbone dynamics, as they did in the paper, the model must be validated to ensure that these components are correctly balanced.

Reply: Thank you very much for raising this important concern regarding the balance of energetic components and overall model validity. We would like to clarify a critical distinction between our MD analysis and the entropy decomposition from Entropy-UNet.

In our MD simulations, the force field partitions the total energy into electrostatic contributions and van-der-Waals (vdW) contributions. Accordingly, the two distinct energetic contributions to the dynamics, classified as electrostatic-type and vdW-type, as shown in Figure 2 (d-e) and (g-h), are technically sound and correctly represented.

In contrast, Entropy-UNet performs a decomposition of entropic contributions, rather than potential-energetic ones. Specifically, it operates on bead-level conformational entropy fingerprints and separately quantifies the entropic contributions from HB, BS, and SPB beads, based on their

spatial positions. Importantly, the summed entropic contributions from stacking and hydrogen-bonding predicted by Entropy-UNet show qualitatively agreement with the MD vdW-type contribution (Figure.4a and Figure.2d). This consistency implies that unwinding dynamics observed from energetic perspective and from spatial distribution perspective are fundamentally aligned.

Based on the authors' observation of an "open-ring configuration" (line 132), I suspect that the stacking interaction may be overestimated. According to experimental evidence, for example Plumridge et al (2020) 10.1021/jacs.9b04461, single-stranded RNA should not exhibit such a perfectly stacked structure. This may also imply that hydrogen bonding is underestimated in the model (so that melting stability is compensated), which could lead to inaccurate unwinding dynamics. For this specific point, the authors could validate the end-to-end distance and/or persistence length of single-stranded RNA.

Reply: As implemented in our HTri-CG model, both base-stacking and hydrogen-bond interactions adopt isotropic 12-10 Lennard-Jones potentials without angular anisotropy. As pointed out by Plumridge et al. (2020, 10.1021/jacs.9b04461), single-stranded RNA does not adopt perfecty stacked conformations. To assess effective stacking strength in our model, we have compared end-to-end-distance distributions of poly(U17) ssRNA between all-atom (Amber99, 1 μs) and coarse-grained simulations (Reply Figure 1). The end-to-end-distance peak is 2.25 nm for all-atom simulation and 2.5 nm for CG simulation. The CG single-strand is even more extended, providing no evidence that the intrachain stacking interaction is overestimated in our model.

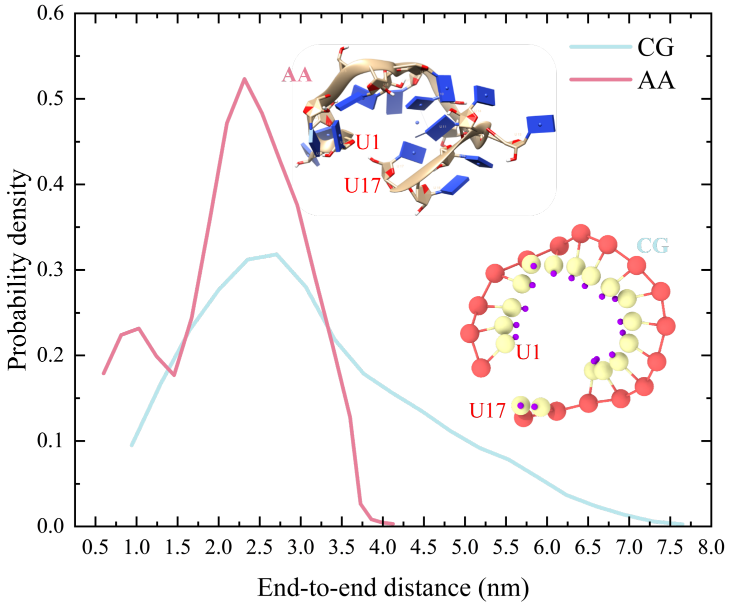

Reply Figure 1. All-atom (AA,amber99 force field, 1 μs) and coarse-grained (CG, 1×10^{9} steps) simulations were conducted for poly (U17). End-to-end distance distributions were derived from the last 100 ns of the all-atom trajectory and the final 1×10^{8} steps of the CG trajectory.

The seemingly well-ordered open-ring snapshots arise from the geometric freedom of the isotropic LJ potential; such ring-like geometries can be sampled with low energetic cost and do not indicate

strong stacking energetics. Furthermore, in our Htri-CG implementation, the base-stacking potential is applied only to bases belonging to the same RNA strand; inter-strand base-stacking interactions are not included (see GitHub: https://github.com/BeastyWK/Entropy-UNet/tree/main/Simulation%20file). This is a simplification of the model, in which duplex stabilization is represented primarily through Watson-Crick hydrogen-bonding interactions. Therefore, dsRNA melting stability is governed purely by Watson-Crick hydrogen-bonding terms. A similar simplification was previously adopted in a coarse-grained DNA model [JCTC 20,1717-1731 (2024)].

We acknowledge that the isotropic Lennard-Jones description of stacking and hydrogen bonding introduces quantitative uncertainties for unwinding kinetic details. Nevertheless, our central physical conclusion that dsRNA unwinding is entropy-driven remains robust. Since all major enthalpic contributions rise during unwinding, the driving force for duplex opening must originate from entropy gain quantified in our work.

The discussion has been revised in the HTri-CG RNA Model section; comparisons of all-atom and coarse-grained end-to-end distances for dsRNA and ssRNA have been added **(S6 Fig.).**

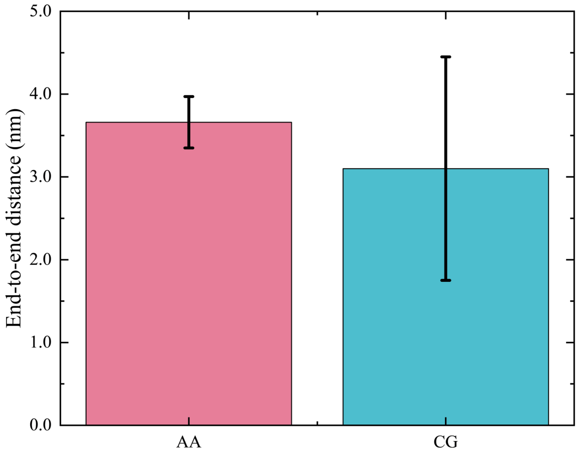

S6 Fig. End-to-end distance comparison between all-atom (GROMACS, amber99, 1 μs, last 100 ns) and CG (total 1x10^9 steps, last 1x10^8 steps) simulation trajectories. PDB ID: 6O5F, 23-bp dsRNA.

The manuscript is revised as follows:

Page 14, Lines 25–31: Notably, the isotropic Lennard-Jones potential used in our coarse-grained model to describe both stacking and hydrogen-bonding interactions tends to produce overly ordered intra-strand stacking conformations. Despite this simplification, quantitative benchmarking against all-atom simulations demonstrates that our model remains applicable to dsRNA. For instance, the averaged end-to-end distance of a 23-bp dsRNA predicted by our model differs from the all-atom results by approximately 15% (S6 Fig.).

In addition, regarding the interaction between RNA and proteins, the authors wrote, (line 163) "We progressively increased the interaction strength between the D1/D2 domains and the Sugar-

Phosphate Bead (SPB) in increments of 0.72ε (where ε is the binding energy in kal/mol) until observable dissociation events occurred. This procedure yielded a measured binding energy of 10.00ε between dsRNA and the D1/D2 domains of DDX3X." --- What does this mean? The interaction parameter between the protein and RNA is particularly important in this study, which focuses on binding and helicase activity. Please could you describe in sufficient detail how the 10.00 \epsilon value was obtained? I do not understand the text above. If you increase the strength, don't you observe association events rather than dissociation?

Reply: Thank you for pointing out this unclear statement. The confusion mainly arose from our use of the term 'dissociation events'. Our original intention was to describe the unwinding of the dsRNA duplex, rather than the dissociation of the D1/D2 domains from the dsRNA. We apologize for the confusion.

Since the experimental binding strength between DDX3X and dsRNA is unavailable, we used a titration-like parameter-scan strategy. At very low attraction, D1/D2 fails to stably bind dsRNA. As the attraction increases, binding becomes possible but duplex unwinding remains inaccessible. Further strengthening the interaction promotes unwinding, although the occurrence of unwinding is stochastic and limited to a relatively narrow window of attraction strengths. Beyond this optimal regime, excessive attraction immobilizes D1/D2 on the duplex and suppresses unwinding by restricting the conformational rearrangements essential for strand separation.

We selected 10.00 kcal/mol within this optimal regime, which yields ~18 % unwinding probability (Figure 2a), qualitatively consistent with experimental findings that ATP-free DDX3X-mediated unwinding is a rare event [Current biology: CB 34, 5714-5727.e5716 (2024)].

We have revised the manuscript text to explicitly state these are dsRNA unwinding events to eliminate misinterpretation.

The manuscript is revised as follows:

Page 5, Lines 23-29: We progressively increased the interaction strength between the D1/D2 domains and the Sugar Phosphate Bead (SPB) in increments of 0.72 ε (where ε is the binding energy in kcal/mol) until observable dsRNA duplex unwinding events were detected. Such unwinding events are stochastic and restricted to a narrow window of attraction strengths. Both insufficient and excessive interactions suppress strand separation. This procedure yielded a working binding energy parameter of 10.00 ε for dsRNA-DDX3X D1/D2 interaction.

(2) The purpose of developing the deep-learning framework Entropy-UNet is still not clear. In the SI, page 6, it is described as a "specialized statistical framework for predicting and decoupling coordination-resolved entropy." However, the coordination-resolved entropy (CRE) is defined as a function of the coordinates. Why, then, is it necessary to "predict" CRE for a given structure using a neural network? CRE should be directly calculable using Eqs. 3–6 in the main text.

Reply: We fully agree that for a single given molecular snapshot, CRE can be deterministically

computed from atomic coordinates via Eqs. 3–6. However, we would like to clarify that Entropy-UNet is not designed as a surrogate to evaluate CRE for an individual snapshot by mimicking Eqs. 3-6. Instead, the network is trained on an ensemble of conformational samples to learn the underlying probability distribution function (PDF) of molecular configurations. The CRE values calculated from Eqs. 3-6 serve as reference ground truth to validate that the PDF learned by the neural network is physically reasonable. Conceptually, this highlights the great utility of machine-learning techniques in statistical physics, since probability distribution functions are central quantities for characterizing thermal fluctuations.

Similarly, for "decoupling", it should also be possible to decompose the coordinate data directly into backbone, HB-bead, and other components. Even more directly, such decomposition could be performed based on the energies, since the coarse-grained model already contains distinct energy terms for stacking, base pairing, and backbone dynamics. I did not fully understand the motivation of using the ML approach here.

Reply: We agree that the energetic contributions can be decoupled because coarse-grained energy is additive, e.g., $U = U_{HB} + U_{BS} + U_{SPB}$. In contrast, the configurational entropy is a function of PDF, which generally cannot be factorized into independent distributions of the HB, BS, SPB coordinates because these degrees of freedom are strongly correlated. Consequently, the entropy cannot, in general, be decomposed into a simple sum of independent contributions from these individual components.

Entropy-UNet, in contrast, is trained on the coupled fingerprint to predict S_{total}. By selectively masking individual channels of this coupled fingerprint, we can quantify the marginal contribution of each interaction channel to the learned entropy landscape. Importantly, this masking-based attribution does not assume an additive decomposition of entropy; rather, it quantifies how the removal of a given interaction-specific feature affects the entropy prediction in the presence of the other coupled degrees of freedom. Such collective contributions are not accessible through a simple coordinate-wise decomposition.

We have further added detailed motivation for this ML approach in the section **Development and Evaluation of Entropy-Unet**.

The manuscript is revised as follows:

Page 10-11, Lines 37-44； 1-4: Artificial Intelligence (AI) has emerged as a powerful tool for extracting features from high-dimensional scientific data. In non-equilibrium dynamics, configurational entropy derives from a joint probability distribution over strongly correlated degrees of freedom. Although CRE can be computed deterministically for individual snapshots, estimating the underlying probability distribution, and elucidating how hydrogen bonding, base stacking, and backbone fluctuations collectively shape the entropic landscape, remains impeded by the non-factorizability of these coupled coordinates. This raises the question of whether AI-based approach can learn entropy-related features from such non-equilibrium trajectories. To explore this, we develop Entropy-UNet, a deep-learning framework designed to learn the conformational distribution of dsRNA directly from simulation trajectories, enabling entropy estimation and

thermodynamic characterization.

(3) How did the authors convert simulation steps to real time? It is claimed that the simulations were performed with a 0.1 ns time step (SI page 5, first paragraph) and accumulated for 100 ms, but if this time step is the actual value used in the numerical integration, the covalent bond potential listed in Table S5 should cause numerical instability. It is often difficult to estimate real-time equivalence in coarse-grained models, particularly when underdamped Langevin dynamics is used. This point should therefore be clarified in the Methods or SI. Alternatively this could be clarified by making LAMMPS input files publicly available.

Reply: In our CG model, RNA and DDX3X are represented at different resolutions: each nucleotide is represented by three beads, whereas the DDX3X protein is described using a substantially coarser sticker-and-spacer representation. The bead mass was therefore parameterized as an effective coarse-grained mass to enable stable integration of the large-scale protein–RNA dynamics. In addition, all reported time values are nominal simulation time computed as the product of simulation steps and integration timestep. No calibration to experimental real-world time was performed.

We appreciate you raising this point and offer two clarifications below:

Regarding the "100 ms" time conversion. The 100 ms referred to in the SI is not the physical time of a single trajectory, but the aggregate sampling time across all 20 REMD replicas. Each replica ran for 5.0×10^{7} steps with a 0.1 ns timestep, yielding 5 ms per replica. The total across 20 replicas is therefore 20 × 5 ms = 100 ms.

Regarding the stability of the 0.1 ns timestep. The bonded interactions in our model are effective CG potentials rather than atomistic covalent-bond potentials. Each coarse-grained bead has an effective mass of 3534.1ag. For example, the RNA bond stiffness of 50 kcal/mol corresponds to approximately 347.4 ag-nm^2/ns^2 in LAMMPS nano units, giving a characteristic bond-vibrational period of approximately 20 ns. Thus, the 0.1 ns timestep provides about 200 integration steps per characteristic bond-vibrational period and does not lead to numerical instability.

Regarding LAMMPS input files. The complete LAMMPS input files have been publicly available on GitHub (https://github.com/BeastyWK/Entropy-UNet/tree/main/Simulation%20file), where the timestep and all other simulation parameters can be verified.

The SI is revised as follows:

(SI Page 5, Lines 20-23): It should be noted that all reported time values are nominal simulation time computed as the product of simulation steps and integration timestep. No calibration to experimental real-world time was performed.

(4) The coarse-grained simulation code (or complete input for LAMMPS), not only the Entropy-UNet code, should be made publicly available. The analysis code would also be valuable for transferability and reproducibility.

Reply: The LAMMPS code is in the github directory: https://github.com/BeastyWK/Entropy-UNet/tree/main/Simulation%20file.

Minor points

- line 47, He et al. (7) - the convention is to use the last name, therefore Song and Ji.

Reply: **Page 2, Line 23 and Page 14 Line 36:** He et al→ Song and Ji.

- line 165, kal/mol

Reply: **Page 5, Line 26:** kal/mol→ kcal/mol.

- line 520, "Calculate single-bead a mollified version"

Reply: **Page 16, Line 13:** Calculate a single-bead smoothed version.
