# Supplementary material for: ATP-independent entropy-driven dsRNA unwinding by DDX3X revealed by coarse-grained simulations and deep learning
Source: PLoS Comput Biol. 2026 Oct 30;22(10):e1014928. doi: 10.1371/journal.pcbi.1014928 (PMC13734570; doi:10.1371/journal.pcbi.1014928)
Supplement: Supplementary file 2 [file pcbi.1014928.s003.pdf]

Reviewers' comments:

Reviewer's Responses to Questions
Comments to the Authors:
Please note here if the review is uploaded as an attachment.

Reviewer #1: Overall, I appreciate that the authors provided a detailed and thoughtful rebuttal. The responses provided in the letter adequately addresses my technical and theoretical concerns. However, I noticed that very few of these substantive explanations were incorporated into the revised manuscript itself. Since future readers will likely not have the benefit of reading the rebuttal letter, these excellent clarifications should be integrated directly into the main text or supplementary information. Looking at the other reviewer responses, I imagine that they would feel similarly (especially reviewer #3 who brought up excellent points), but I will focus on my own review here.

1) The authors provide a strong explanation for how their HelixTriad model builds on the three-bead model by decoupling base-stacking and hydrogen bond interactions and sets the foundation for protein-RNA interactions. It seems natural to add this rationale in the introduction or method section to clarify the model's advancement over previous iterations, namely the Hyeon and Thirumalai, Denesyuk and Thirumalai, and Kapoor, Kim, and Mittal three-bead models.

Reply: We agree and have added this rationale to the "Development and Validation of the HTri-CG Model" section.

**The manuscript is revised as follows:**
**Page 4, Lines 3-12:** The three-bead-per-nucleotide framework has a long history. Hyeon and Thirumalai introduced explicit base stacking into a three-bead RNA model[23]. Denesyuk and Thirumalai subsequently added an explicit directional hydrogen-bonding potential, but both stacking and hydrogen bonding acted on the same base bead and thus could not be physically decoupled[24]. Recently, Kapoor, Kim, and Mittal adopted a three-bead DNA representation with distinct phosphate-sugar, base, and hydrogen-bonding sites[16]. Building on this idea, we built upon a three-bead-per-nucleotide framework, and parameterized three distinct interaction sites to achieve physical separation of hydrogen bonding, base stacking and phosphodiester backbone electrostatics, yielding HelixTriad coarse-grained (HTri–CG) RNA model (Fig.1a).

2) The authors make a fairly bold prediction from their model about the previously unrecognized physical mechanism for RNA duplex unwinding. I think that the suggestions for future experimental validation using single molecule tools like deFCCS and smFRET-anisotropy to detect transient partially unwound intermediates are excellent. It would benefit this manuscript to integrate this discussion into the Discussion or Conclusion to actively guide future in vitro testing.

Reply: We thank Reviewer for this suggestion. We have added a paragraph to the Discussion and Conclusion to make the experimental implications of our model explicit and testable.

**The manuscript is revised as follows:**

**Page 14, Lines 10-23:** A key prediction of the proposed entropy-driven mechanism is that DDX3X can promote rare, transient strand-displaced intermediates even in the absence of ATP. This prediction can be tested on short, low-stability dsRNA substrates under ATP-free conditions, with particular attention to detecting transient PU intermediates and their occurrence probabilities. Several relevant experimental approaches have already been applied to this system. Dual-color fluorescence cross-correlation spectroscopy (dcFCCS), single-molecule FRET and fluorescence anisotropy, and mass photometry have resolved DDX3X RNA-protein cluster formation, RNA capture, and strand release in vitro[12]. Complementing these approaches, site-specific $^{19}$F NMR ribose probes can report on local and global dsRNA-ssRNA interconversion with minimal interference from protein background; previous studies using such probes have shown that DDX3X can bind a locally unwound duplex and adopt an ssRNA-bound closed conformation[10]. Together, these complementary approaches provide a promising route to directly test key predictions of the stochastic, entropy-driven unwinding mechanism proposed here.

Reviewer #2: The authors have almost fully addressed my concerns in the last round review. Now I recommend its acceptance.

Reply: We sincerely thank Reviewer for the positive recommendation and for the helpful comments throughout the review process.

Reviewer #3: I appreciate that the authors have addressed most of the reviewer comments in the revised manuscript and rebuttal. Although I still have some reservations about the quantitative accuracy of the RNA coarse-grained model, I also acknowledge that the authors have described the model in very detail, and that the simulation code, parameters, and relevant implementation details have been made openly available. Taking these revisions into account, and considering that the manuscript now provides sufficient methodological detail and open resources for readers to assess the validity and limitations of the model, I think the manuscript may be suitable for publication.

However, I would like to record one remaining point of disagreement with the authors' rebuttal. In response to concerns about the behaviour of single-stranded RNA in their coarse-grained model, the authors compared the end-to-end distance obtained from their coarse-grained simulations with that from Amber atomistic MD simulations. In my view, this comparison does not validate the coarse-grained force field. Atomistic MD simulations should not be treated as a ground truth for RNA conformational ensembles. In particular, it is known that Amber RNA force fields can substantially overestimate compact conformations of single-stranded RNA. For example, see Fig. 3C of Tan et al., PNAS 2018, 10.1073/pnas.1713027115.

This is a common misconception. Atomistic MD simulations are not always correct, particularly for RNA. Since experimental data are available for simple ssRNA systems such as poly(A) and poly(U), validation of single-stranded RNA behaviour should ideally be performed against experimental observables rather than only against Amber simulations.

Although the authors discussed this ssRNA comparison in the rebuttal, the revised manuscript appears to include only a dsRNA comparison and not the ssRNA simulation result. Therefore, I do not request further revision regading this.

Reply: We thank the reviewer for this important clarification. We agree that atomistic MD simulations should not be regarded as ground truth for RNA conformational ensembles, particularly for single-stranded RNA, for which force-field-dependent biases have been reported. Our comparison with Amber simulations was intended as a reference comparison rather than as definitive validation of the coarse-grained force field. We acknowledge that validation against experimental observables, such as those available for poly(A) and poly(U), would provide a more direct assessment of ssRNA conformational behaviour. We appreciate the reviewer's thoughtful comments and the recognition that the revised manuscript provides detailed methodological information and openly available simulation resources for assessing the model and its limitations.
