# Supplementary material for: ATP-independent entropy-driven dsRNA unwinding by DDX3X revealed by coarse-grained simulations and deep learning
Source: PLoS Comput Biol. 2026 Oct 30;22(10):e1014928. doi: 10.1371/journal.pcbi.1014928 (PMC13734570; doi:10.1371/journal.pcbi.1014928)
Supplement: Supplementary file 3 [file pcbi.1014928.s001.docx]

**Supplementary Information**

**ATP-Independent Entropy-Driven dsRNA Unwinding by DDX3X Revealed by Coarse-Grained Simulations and Deep Learning**

Kang Wang^1^, Chun-Lai Ren^1,2,*^, &Yu-Qiang Ma^1,2^

^1^National Laboratory of Solid State Microstructures and Department of Physics,

Collaborative Innovation Center of Advanced Microstructures, Nanjing University, Nanjing, China

^2^Hefei National Laboratory, Hefei, China

*chunlair@nju.edu.cn (C.-L. R.)

**Contents**

**Supplementary Text**

1.1 The Coarse-Grained Modelling

1.2 Metadynamics Implementation

1.3 Replica exchange molecular dynamics Implementation

1.4 dsRNA and Hairpin constructs

1.5 Length-scale mapping

1.6 Entropy-Unet network architecture

1.7 Supplementary Figures

1.8 Supplementary Tables

**1 Supplementary Text**

**1.1 The Coarse-Grained Modelling**

The HTri-CG RNA model employs a three-bead-per-nucleotide representation comprising: (i) Base stacking interactions site beads, (ii) WC base pairing hydrogen bonding site beads, and (iii) sugar-phosphate backbone interactions site beads. Systematic mass parameterization of these coarse-grained units (each bead has an effective mass of 3534.1ag) was implemented to facilitate microsecond-scale molecular dynamics simulations while preserving RNA structural fidelity. The base beads are classified into four distinct types: adenine (ADE, A), uracil (URA, U), cytosine (CYT, C), and guanine (GUA, G). Base stacking interactions were systematically categorized into 10 discrete classes (A-A, A-U, A-C, A-G, U-U, U-C, U-G, C-C, C-G, G-G), governed by a 12-10 Lennard-Jones (12-10 LJ) potential (Eq.7). Using the high-level quantum chemical stacking energies from Mignon et al^1^., as a foundation, we applied the Lorentz-Berthelot mixing rules to parameterize these interactions, including cross-base pairs; the complete parameter set is detailed in Table B. Nonbonded Interaction Parameters for Base Stacking Interactions. This model strictly enforces canonical WC base pairing, with G forming exclusive hydrogen bonds with C, and A pairing specifically with U. In our simulation, base-stacking interactions involving non-canonical bases are fully preserved, while only hydrogen-bonding pairing terms for such bases are disabled. This treatment is motivated by the physical consideration that, for the short dsRNA, inter-strand interactions are expected to diminish rapidly as the double helix dissociates, whereas intra-strand interactions can be preserved. Analogous to base stacking interactions, a 12-10 LJ potential was implemented. WC base pair hydrogen-bonding parameters were parameterized using equations and tabulated values provided in Eq.8 and Table C. Nonbonded Interaction Parameters for Hydrogen Bonding Interactions., respectively. For the SPB, which were assigned -1 charges, electrostatic interactions were modeled using the Debye-Hückel potential as detailed in SI Eq.11.

The coarse-grained modeling of DDX3X incorporates critical molecular features demonstrated in prior studies^2^: The IDRs at both termini exhibit similar residue distributions, with N- and C-terminal segments showing high densities of cationic residues (arginine and lysine) and aromatic residues (phenylalanine and tyrosine). Based on this molecular architecture, we assigned cationic residues as charged (C) beads, aromatic residues as π-interaction (π) beads, and glutamate residues as spacer (S) beads to computationally IDRs behavior of DDX3X. The IDRs were discretized according to their native length ratios, with IDR1 (N-terminal) and IDR2 (C-terminal) segmented in a 2.4:1 proportion (S6 Fig.). This coarse-graining scheme produced 24 beads for IDR1 (composition: 4C, 4π, 16S beads) versus 10 beads for IDR2 (1C, 1π, 8S beads). The catalytic core was constructed by bond linking D1 and D2 domain beads, resulting in a 36-bead protein chain representing the complete DDX3X architecture. Based on Song and Ji findings^3^, the D1/D2 domains predominantly engage with RNA's phosphate backbone and ribose moieties (represented as SPB beads in HTri-CG RNA). We therefore model the DDX3X-dsRNA interaction as specific bead-bead interactions between D1/D2 beads and SPB beads, employing a soft potential with a 14 nm cutoff distance. Detailed parameters and mathematical formulations are provided in Table D. Nonbonded Interaction Parameters for Sticker and Eq.9. For complete details of the coarse-grained modeling.

The DDX3X-dsRNA interaction in our model framework specifically incorporates two designed components: (i) electrostatic attraction between the D1/D2 domains and the negatively charged sugar-phosphate backbone, coupled with (ii) steric repulsion inherent to the backbone bead architecture. This dual-mechanism design explicitly captures both domain-specific binding and non-bonded excluded volume effects.

The total potential energy of the system comprises three principal components,$\begin{aligned} U_{total}=U_{RNA-total}+U_{Protein-total}+U_{RNA-Protein}\#(1) \end{aligned}$

where the Protein energy term *U_Protein-total_* is decomposed as:

$$\begin{aligned} U_{\mathrm{Protein}-total}=U_{\mathrm{bond}}+U_{\mathrm{sticker}}+U_{excluded}\#(2) \end{aligned}$$

Similarly, the RNA energy term *U_RNA-total_* is decomposed as:

$$\begin{aligned} U_{RNA-total}=U_{bond}+U_{angle}+U_{stack}+U_{HB}+U_{excluded}+U_{electrostatics}\#(3) \end{aligned}$$

Cross-component interactions *U_RNA-protein_* between RNA and protein are governed by specific sticker interactions and steric exclusion:

$$\begin{aligned} U_{RNA-protein}=U_{sticker}+U_{excluded}\#(4) \end{aligned}$$

Where bonded interactions are modeled using harmonic potentials with equilibrium distances:

$U_{bond}=\sum_{i}^{N_{bond}} K_{bond}[r-r_{b}]^{2}$	(5)

And the Backbone−backbone−backbone angular, Backbone−base−HB side chain angular constraints employ cosine-harmonic potentials to maintain RNA structural topology:

$U_{angle}=\sum_{i}^{N_{angle}} K_{angle}[\cos\theta-\cos\theta_{0}]^{2}$	(6)

Complete parameterization details are provided in Table E. Bonded Interaction parameters related to Bond and Angle.

The *U_stack_* potential governs base stacking interactions, operating exclusively as an intramolecular force restricted to sequentially adjacent residues (*i*, *i*+1) within individual strands. This orientation-specific potential is implemented through a 12-10 Lennard-Jones (LJ), maintaining structural integrity independent of base-pairing configurations:

$$\begin{aligned} U_{stack}&=\sum_{i<j}^{N_{stack}} \left\{ \begin{matrix} \delta_{stack}\left[ 5\left( \frac{\sigma_{stack}}{r} \right)^{12}-6\left( \frac{\sigma_{stack}}{r} \right)^{10} \right], & if r\leq r_{stack} & & & \\ & & & & (7) \\ 0, & otherwise & & & \end{matrix} \right. \end{aligned}$$

Complete parameterization details of base stacking are provided in Table C. Nonbonded Interaction Parameters for Hydrogen Bonding Interactions.

The *U_HB_* potential governs Watson-Crick hydrogen bonding interactions, operating both intramolecularly and intermolecularly. This directional potential is selectively disabled for consecutive HB triplets (positions i, i+1, i+2) within individual strands. Implemented through a 12-10 LJ analogous to the stacking potential, it specifically mediates canonical base-pair hydrogen bonding recognition.

$U_{HB}=\sum_{basepairs}^{N_{bp}} \left\{ \begin{aligned} \delta_{HB}\left[ 5\left( \frac{\sigma_{HB}}{r} \right)^{12}-6\left( \frac{\sigma_{HB}}{r} \right)^{10} \right], if r\leq r_{HB} \\ & 0, otherwise&& \end{aligned} \right.$ (8)

Complete parameterization details of hydrogen bonding are provided in Table D. Nonbonded Interaction Parameters for Sticker.

The *U_sticker_* orchestrates two mechanistically distinct processes:(i) Cation-π-driven coacervation between Cationic and aromatic residues, which governs liquid-liquid phase separation (LLPS) nucleation through valency-dependent transient polypeptide assemblies; (ii) specific D1/D2 domain engagements with SPB, constituting the mechanistic basis for ATP-independent or ATP-dependent dsRNA unwinding activity^2^.

$U_{sticker}=\sum_{sticker}^{N_{sticker}} \delta_{sticker}\left( 1+cos\frac{\pi r}{r_{sticker}} \right),r<r_{sticker}$	(9)

Complete parameterization details of sticker interactions are provided in Table E. Bonded Interaction parameters related to Bond and Angle.

The *U_excluded_* potential enforces steric exclusion through implementation of the Weeks-Chandler-Andersen (WCA) truncated repulsive potential, where the energy scale parameter (*ε_ex_*) modulates exclusion intensity.

$U(r)_{excluded}=\sum_{i<j}^{N_{ex}} \left\{ \begin{aligned} & 4\varepsilon_{ex}\left[ \left( \frac{\sigma}{r} \right)^{12}-\left( \frac{\sigma}{r} \right)^{6} \right]+\varepsilon_{ex} if r\leq2^{1/6}\sigma_{ex} \\ & 0, otherwise&& \end{aligned} \right.$	(10)

RNA CG beads utilize enhanced steric exclusion with *ε_ex_* = 27.788*ε* and σ= 0.55 nm, while protein CG beads adopt reduced repulsion parameters *ε_ex_* = 5.0*ε* and σ= 0.55 nm. Notably, hydrogen-bonding competent beads are explicitly exempted from steric exclusion constraints to permit directional bonding geometry.

Lastly, the *U_electrostatics_* potential governs electrostatic interactions between uniformly charged sugar-phosphate backbone beads, which have an overall -1 charge. These long-range interactions are modeled via the Debye-Hückel approximation:

$U_{\text{electrostatics}}=\sum_{i<j}^{N_{backbone}} \frac{q_{i}q_{j}}{4\pi\varepsilon_{0}\varepsilon_{r}r_{ij}}e^{-r_{ij}/\kappa}$	(11)

*ε_0_* corresponds to the vacuum dielectric permittivity, *ε_r_* denotes the relative dielectric constant of water (set to 80). and *κ* is the Debye screening length, which is taken as 10, 7.95, 3.0 and 1.54 for 100, 150, 1000 and 4000 mM salt concentrations, respectively. To accelerate the computational efficiency, a cutoff distance of 3.5*κ* is employed.

**1.2 Metadynamics Implementation**

To more efficiently investigate the statistical behavior of the DDX3X-dsRNA complex system, we employed both MD simulations and metadynamics simulations. Metadynamics constitutes an enhanced sampling methodology wherein a history-dependent bias potential, composed of sequentially deposited Gaussian functions, is applied along predefined collective variables (CVs)^4^. To elucidate the DDX3X-dsRNA dynamics at 300 K within a cubic periodic boundary box(30³nm³), we conducted metadynamics simulations using the radius of gyration of the entire system (Rg^system^, range 0–6.0 nm) and the center-of-mass distance between two strands of the dsRNA (Dist, range 0–3.0 nm) as CVs, respectively. The Rg^system^ CV monitored the conformational compactness of DDX3X-dsRNA complex, utilizing a 0.14 kcal/mol Gaussian weight with a 5.7 nm harmonic constraint (2.87 kcal/mol/nm²). The Dist CV was biased using metadynamics parameters: 0.09 kcal/mol Gaussian hill potential, 1240 K bias temperature, and a 2.87 kcal/mol/nm² harmonic wall restraint at 27 nm to constrain maximal separation. Both variables employed well-tempered metadynamics in “COLVARS” module of LAMMPS^5^, depositing new hills every 1,000 steps and writing free energy profiles at 500,000-step intervals. The simulations probed DDX3X helicase-mediated dsRNA unwinding, reconstructing the potential of mean force (PMF) along the dissociation pathway. Concurrently, the accumulated conformational data (4.0×10^4^ frames per CVs dimension) provided a robust dataset for training neural networks to predict Coordination-resolved entropy of dsRNA.

**1.3 Replica exchange molecular dynamics Implementation**

To validate the suitability of HTri-CG RNA for quantitative studies of dsRNA unwinding process, we first employed this model to investigate the thermal melting of dsRNA. The stability of duplex is primarily governed by both strand concentration and length, largely due to the translational entropy contributed by f a new kinetic unit on duplex dissociation. However, at low strand concentrations (e.g., ~ 0.1mM in experiments), extended simulation times are generally necessary to attain system equilibrium. To improve sampling efficiency under these conditions, we employed REMD simulations^6^. The simulations use two distinct dsRNA systems: a short 14-bp duplex (S1S2, 28-nt) and a long 23-bp duplex with interloop elements (XY, 73-nt). The simulation protocol involved randomly distributing ten copies of each dsRNA variant in a cubic box (30×30×30 nm³). The ten copies are independent of each other. REMD simulations were conducted with 20 replicas spanning 290-370 K using the LAMMPS package (August 2024 version). The coarse-grained simulations employed Langevin dynamics in the NVT ensemble, utilizing a Langevin thermostat with a damping coefficient equivalent to 1000. Each replica underwent 5.0×10^7^ steps with a 0.1ns timestep, yielding an aggregate sampling time of 100ms across all replicas. It should be noted that all reported time values are nominal simulation time computed as the product of simulation steps and integration timestep. No calibration to experimental real‑world time was performed. Configuration exchanges between replicas were attempted at 100-step intervals, with structural snapshots recorded every 10^5^ steps. After discarding the initial 0.5ms as equilibration, trajectory analysis was performed on the remaining 4.5ms of production data. Melting profiles were constructed by analyzing 20 representative snapshots from each replica, generating a total ensemble of 4,000 conformations for statistical analysis.

**1.4 dsRNA and Hairpin constructs**

In this subsection, we selected the dsRNA and hairpin RNA employed for parameterizing and validating the HTri-CG RNA model. The primary validation system utilized a dsRNA consisting of 14 base pairs (28 nucleotides in total; S1: 5′-CCUUGAUAUCAAGG-3′; S2:5′-GGAACUAUAGUUCC-3′; see Fig.1a). Additionally, a 73-nucleotide dsRNA with 23 base pairs and two interloops (X: 5'-UCAGGCGUAACUCAAAGCCCCUUCUCAGGCAAGCAC-3';Y:5'-CUCGGGAUCCGAGAAGGAAAAAGCUUCUACGCCUGAA-3'; see Fig.1b) was employed. These molecules were chosen due to the availability of complementary computational and experimental melting curves, which facilitated comparative analysis. For tertiary validation, a 5-bp stem tetraloop hairpin (5′-GGCACUUCGGUGCC-3′, see Fig.1c) was specifically chosen as a test case of RNA flexibility. This selection criterion stems from our previous findings showing that shorter 3-bp hairpin structures exhibit prohibitive instability for reliable validation^7^. Our validation paradigm establishes two critical boundary conditions: (1) Under excessive strand flexibility, the tetra-loop hairpin would maintain artificial stability throughout prolonged simulations, and (2) Hyper-rigidity would induce irreversible structural collapse into disordered states. Valid parameterization requires intermediate flexibility enabling transient unraveling followed by spontaneous reformation into the native 5-bp configuration.

**1.5 Length-scale mapping**

All simulations were performed using the LAMMPS nano unit system, where distance, time, and energy are expressed in nanometers (nm), nanoseconds (ns), and attogram-nanometer^2^/nanosecond^2^, respectively. These simulation units define the numerical integration framework but do not imply a direct one-to-one mapping to atomistic dimensions. Because RNA and DDX3X were represented at different coarse-graining resolutions, we employed a heterogeneous coarse-graining strategy and calibrated the fundamental length scale using RNA structural properties. Specifically, the RNA model was parameterized to reproduce experimentally measured duplex properties, including helix pitch, groove width, and persistence length. To compare simulated RNA structures with experimental measurements, a constant length-rescaling factor λ = 0.1 was introduced:

*l*_phys_ = λ *l*_sim_

where *l*_sim_ is the simulation length in LAMMPS nano units and *l*_phys_ is the corresponding physical length. Under this mapping, 10 simulation nanometers correspond to 1 physical nanometer.

**1.6 Entropy-Unet network architecture**

The coarse-grained molecular dynamics simulations produce extensive conformational ensembles whose high-dimensional characteristics and analytical complexity have resulted in systematic underutilization within computational biophysics research. In our methodology, deep learning is strategically implemented as a specialized statistical framework for predicting and decoupling coordination-resolved entropy through systematic feature extraction from these conformational datasets. This study employed two benchmark datasets constructed using metadynamics simulations under distinct reaction coordinates: (i) The Rg^system^ coordinate system underwent 4.0 × 10^9^ enhanced sampling steps (0.1 ns/step) for DDX3X-dsRNA complex conformational exploration, producing 40,000 conformations; (ii) The Dist served as the second coordinate system, employing identical sampling parameters to generate an equivalent number of conformations. Given the substantial conformational overlap observed during the initial equilibration phases of both simulations, we excluded 10,000 configurations from the preliminary phase of the second dataset. The final composite dataset comprised 70,000 distinct conformations randomly divided into training, validation, and testing sets in an 8:1:1 ratio.

The Entropy-Unet architecture employs a specialized encoder-decoder framework for probabilistic predicting of coordination number of beads in dsRNA (see Fig.4b, P*). The encoder module progressively abstracts hierarchical features through four downsampling stages (2×2 max pooling), each comprising two convolution blocks (3×3 kernels, ReLU activation) that extract localized transient thermal fluctuations of dsRNA. Conversely, the decoder module reconstructs coordination probability distributions via transpose convolutions (2×2 stride), with skip connections enabling feature concatenation between corresponding encoder-decoder levels to preserve entropy correlations. The overall design follows the U-Net framework^8^, a widely-used convolutional neural network in biomedical image segmentation.

The Entropy-Unet is optimized through minimization of the Kullback-Leibler Divergence (KLD) between the predicted log probability distribution P* and the truth probability distribution P, employing the Adam optimizer with learning rate=0.001. Probability normalization is achieved through the softmax function activation in the final output layer. The loss function as below.

$$\begin{aligned} Loss\left( P,P^{*};\theta\right)=\frac{1}{N}\sum_{i=1}^{N} \sum_{j=1}^{C} P_{ij}\left( logP_{ij}-P_{ij}^{*} \right)\#\left( 13 \right) \end{aligned}$$

Where θ=8,644,485 is used to represent all parameters in the neural network, N is the batch size, and C is the number of beads. The training process was quantitatively assessed using four complementary metrics: KLD Loss and Cross-Entropy (CE) Loss for probabilistic calibration, Brier Score (BS) for prediction accuracy, and Cosine Similarity (CS) for directional consistency analysis.

$$KLD\left( P,P^{*} \right)=\frac{1}{N}\sum_{i=1}^{N} \sum_{j=1}^{C} P_{ij}\left( \log P_{ij}-P_{ij}^{*} \right) (14)$$

$$CE\left( P,P^{*} \right)=-\frac{1}{N}\sum_{i=1}^{N} \sum_{j=1}^{C} \log\left( P_{ij} \right)*P_{ij}^{*} (15)$$

$$BS(P,P^{**})=\frac{1}{N}\sum_{i=1}^{N} \sum_{j=1}^{C} (P_{ij}-P_{ij}^{**})^{2} (16)$$

$$CS(P,P^{**})=\frac{1}{N}\sum_{i=1}^{N} \frac{P_{i}*P_{i}^{**}}{\parallel P_{i}\parallel*\parallel P_{i}^{**}\parallel} (17)$$

where N=70 is the batch size, and C=84 is the number of beads, P is ground-truth coordination number distributions of beads, P* is log probability distributions by Entropy-Unet predicted. P** is probability distributions by postprocessing.

**Supplementary Figures**

Fig A. Predication of dsRNA melting curves. (a) The *T_m_* of the 10 S1S2 dsRNA and the 6 dsRNA are 349.5 K and 350.0 K, respectively. (b) The *T_m_* of the 10 XY dsRNA and the 6 dsRNA are 336.5 K and 337.8 K, respectively.

Fig B. The states of IDRs of DDX3X during unwinding.

Fig C. The pathway dsRNA unwinding by DDX3X.

Fig D. Grad-CAM for HB, BS and SPB Fingerprints. (a)1.35×10^9^ step (open 3-bp) in group 6 of 15³nm³ box. (b)1.6×10^9^ step (open 6-bp) in group 6 of 15³nm³ box. (c)1.4×10^9^ step (open 4-bp) in group 1 of 15³nm³ box.

Fig E. The CRE computed by CNN,Theoretical calculations (CRE-TC), Entropy- UNet, Transfomer-Encoder.

Fig F. End‑to‑end distance comparison between all‑atom (GROMACS, amber99, 1 μs, last 100 ns) and CG (total 1x10^9^ steps, last 1x10^8^ steps) simulation trajectories. PDB ID: 6O5F, 23‑bp dsRNA.

Fig G. Schematic diagram (not to scale) of the DDX3X model architecture. TOP: a linear diagram of the features of the DDX3X protein. Bottom: The DDX3X molecular components Cationic(C) and Aromatic(π) residues were structurally represented as fluorescent and green spherical beads, respectively, while functional domains were depicted through white (D1 bead=NET+D1) and goose-yellow (D2 bead=D2+CET) spheres. with intervening Spacer regions illustrated as beige S bead.

**Supplementary Tables**

Table A. Structural parameters for A-RNA helices^9^.

| Structural property | Ideal A-dsRNA(Å) | Htri-CG RNA (Å) |
| --- | --- | --- |
| Base rise | 2.8 | 3.24 ± 0.54 |
| Helical width | 23.3 | 22.66 ± 0.67 |
| Base pairs per turn | 10.7–11.0 | 11.40 ± 1.60 |
| Major groove width | 4 | 4.65 ± 0.96 |
| Minor groove width | 11 | 12.35 ± 0.49 |

Table B. Nonbonded Interaction Parameters for Base Stacking Interactions

| Base Type | *r_stack_* | *σ_stack_* | *δ_stack_(ε)* |
| --- | --- | --- | --- |
| A−A | 6.2 | 3.3 | 8.82 |
| A−U | 6.2 | 3.3 | 9.90 |
| A−C | 6.2 | 3.3 | 9.50 |
| A−G | 6.2 | 3.3 | 11.17 |
| U−U | 6.2 | 3.3 | 6.35 |
| U−C | 6.2 | 3.3 | 8.52 |
| U−G | 6.2 | 3.3 | 10.63 |
| C−C | 6.2 | 3.3 | 8.26 |
| C−G | 6.2 | 3.3 | 9.32 |
| G−G | 6.2 | 3.3 | 11.32 |

Table C. Nonbonded Interaction Parameters for Hydrogen Bonding Interactions.

| HB Type | *r_HB_* | *σ_HB_* | *δ_HB_(ε)* |
| --- | --- | --- | --- |
| A−U | 3.3 | 1.65 | 8.74 |
| G−C | 3.3 | 1.65 | 9.89 |

Table D. Nonbonded Interaction Parameters for Sticker

Interactions

| Sticker | *r_sticker_* | *δ_sticker_(**ε)* |
| --- | --- | --- |
| C−π | 5.0 | -4.00 |
| D1−SPB | 14.0 | -10.00 |
| D2−SPB | 14.0 | -10.00 |

Table E. Bonded Interaction parameters related to Bond and Angle.

|  | Type | *K_bond_* or *K_angle_*(*ε*) | *r_b_* or *θ* |
| --- | --- | --- | --- |
| Bonds | Backbone−backbone | 50 | 5.5 |
|  | Backbone−base | 50 | 5.75 |
|  | Base−HB | 50 | 1.95 |
|  | Protein Backbone−backbone | 10 | 5.5 |
| Angles | Backbone−backbone−backbone | 20 | 180° |
|  | Backbone−base−HB | 80 | 180° |

**References**

1.	Mignon P, Loverix S, Steyaert J, Geerlings P. Influence of the pi-pi interaction on the hydrogen bonding capacity of stacked DNA/RNA bases. *Nucleic acids research* **33**, 1779-1789 (2005).

2.	Yanas A, Shweta H, Owens MC, Liu KF, Goldman YE. RNA helicases DDX3X and DDX3Y form nanometer-scale RNA-protein clusters that support catalytic activity. *Current biology : CB* **34**, 5714-5727.e5716 (2024).

3.	Song H, Ji X. The mechanism of RNA duplex recognition and unwinding by DEAD-box helicase DDX3X. *Nature communications* **10**, 3085 (2019).

4.	Chattaraj A, Shakhnovich EI. Separation of sticker-spacer energetics governs the coalescence of metastable condensates. *Biophysical journal* **124**, 428-439 (2025).

5.	Lammps. https://github.com/lammps/lammps/tree/stable_29Aug2024_update2.

6.	Zhou R. Replica exchange molecular dynamics method for protein folding simulation. *Methods in molecular biology (Clifton, NJ)* **350**, 205-223 (2007).

7.	Wang K*, et al.* Effect of pausing on the cotranscriptional folding kinetics of RNAs. *International journal of biological macromolecules* **221**, 1345-1355 (2022).

8.	Ronneberger O, Fischer P, Brox TJA. U-Net: Convolutional Networks for Biomedical Image Segmentation. **abs/1505.04597**, (2015).

9.	Ruszkowska A, Ruszkowski M, Hulewicz JP, Dauter Z, Brown JA. Molecular structure of a U•A-U-rich RNA triple helix with 11 consecutive base triples. *Nucleic acids research* **48**, 3304-3314 (2020).
