# Supplementary material for: ATP-independent entropy-driven dsRNA unwinding by DDX3X revealed by coarse-grained simulations and deep learning
Source: PLoS Comput Biol. 2026 Oct 30;22(10):e1014928. doi: 10.1371/journal.pcbi.1014928 (PMC13734570; doi:10.1371/journal.pcbi.1014928)
Supplement: Peer review history

# Peer review: Decision Letter 0

**DOI:** 10.1371/journal.pcbi.1014928.r001

**Authors:** Shi-Jie Chen, Dirk Walther

27 Aug 2026

ATP-Independent Entropy-Driven dsRNA Unwinding by DDX3X Revealed by Coarse-Grained Simulations and Deep Learning

PLOS Computational Biology

Dear Dr. Ren,

Thank you for submitting your manuscript to PLOS Computational Biology. After careful consideration, we feel that it has merit but does not fully meet PLOS Computational Biology's publication criteria as it currently stands. Therefore, we invite you to submit a revised version of the manuscript that addresses the points raised during the review process.

Please submit your revised manuscript by Oct 26 2026 11:59PM. If you will need more time than this to complete your revisions, please reply to this message or contact the journal office at ploscompbiol@plos.org. When you're ready to submit your revision, log on to https://www.editorialmanager.com/pcompbiol/ and select the 'Submissions Needing Revision' folder to locate your manuscript file.

Please include the following items when submitting your revised manuscript:

* A letter that responds to each point raised by the editor and reviewer(s). You should upload this letter as a separate file labeled 'Response to Reviewers'. This file does not need to include responses to formatting updates and technical items listed in the 'Journal Requirements' section below.

* A marked-up copy of your manuscript that highlights changes made to the original version. You should upload this as a separate file labeled 'Revised Manuscript with Track Changes'.

* An unmarked version of your revised paper without tracked changes. You should upload this as a separate file labeled 'Manuscript'.

We look forward to receiving your revised manuscript.

Kind regards,

Shi-Jie Chen

Academic Editor

PLOS Computational Biology

Dirk Walther

Section Editor

PLOS Computational Biology

**Additional Editor Comments (if provided):**

**Journal Requirements:**

If the reviewer comments include a recommendation to cite specific previously published works, please review and evaluate these publications to determine whether they are relevant and should be cited. There is no requirement to cite these works unless the editor has indicated otherwise.

1) Please ensure that the CRediT author contributions listed for every co-author are completed accurately and in full.

At this stage, the following Authors/Authors require contributions: Kang Wang, Chun-Lai Ren, and Yu-Qiang Ma. Please ensure that the full contributions of each author are acknowledged in the "Add/Edit/Remove Authors" section of our submission form.

The list of CRediT author contributions may be found here: https://journals.plos.org/ploscompbiol/s/authorship#loc-author-contributions

2) We ask that a manuscript source file is provided at Revision. Please upload your manuscript file as a .doc, .docx, .rtf or .tex. If you are providing a .tex file, please upload it under the item type u2018LaTeX Source Fileu2019 and leave your .pdf version as the item type u2018Manuscriptu2019.

3) Please provide an Author Summary. This should appear in your manuscript between the Abstract (if applicable) and the Introduction, and should be 150-200 words long. The aim should be to make your findings accessible to a wide audience that includes both scientists and non-scientists. Sample summaries can be found on our website under Submission Guidelines:

https://journals.plos.org/ploscompbiol/s/submission-guidelines#loc-parts-of-a-submission

4) Please upload all main figures as separate Figure files in .tif or .eps format. For more information about how to convert and format your figure files please see our guidelines:

https://journals.plos.org/ploscompbiol/s/figures

5) Please upload a copy of Figure Fig 1-4 which you refer to in your text on pages 3, 5, 9, and 10. Or, if the figure is no longer to be included as part of the submission please remove all reference to it within the text.

6) We have noticed that you have uploaded Supporting Information files, but you have not included a list of legends. Please add a full list of legends for your Supporting Information files after the references list.

7) Please amend your detailed Financial Disclosure statement. This is published with the article. It must therefore be completed in full sentences and contain the exact wording you wish to be published.

i) State the initials, alongside each funding source, of each author to receive each grant. For example: "This work was supported by the National Institutes of Health (####### to AM; ###### to CJ) and the National Science Foundation (###### to AM)."

ii) State what role the funders took in the study. If the funders had no role in your study, please state: "The funders had no role in study design, data collection and analysis, decision to publish, or preparation of the manuscript."

iii) If any authors received a salary from any of your funders, please state which authors and which funders..

If you did not receive any funding for this study, please simply state: u201cThe authors received no specific funding for this work.u201d

**Reviewers' comments:**

Reviewer's Responses to Questions

**Comments to the Authors:**

**Please note here if the review is uploaded as an attachment.**

Reviewer #1: Overview:

Wang et al. investigate the physical mechanism behind the ATP independent double-stranded RNA unwinding by the DEAD-box helicase DDX3X. The authors introduce a three-bead per nucleotide coarse-grained model (HelixTriad) that incorporates base-stacking, hydrogen bonding, and backbone electrostatics that is similar to those previously developed in the Thirumalai group over the past 20+ years. Using a combination of Langevin dynamics and metadynamics, they observe that DDX3X-mediated unwinding is an inherently stochastic, entropy-driven process that overcomes the net positive enthalpy barrier through thermal fluctuations and conformational entropy gain. The authors also develop a U-Net-based deep learning architecture (Entropy-UNet) to predict coordination-resolved entropy (CRE) from “soft pixel fingerprints. Using the feature-attribution analysis, they rank thermodynamic factors driving unwinding: hydrogen bonding > base stacking > backbone electrostatics.

The manuscript is generally well-organized and combines classical statistical mechanics with modern deep learning architecture to address an intriguing biophysical question. However, there are several methodological and analytical issues that must be addressed prior to publication:

Major Comments:

1) The authors state that their HelixTriad coarse-grained model is an extension of previous three-bead-per-nucleotide frameworks proposed in previous studies by explicitly incorporating hydrogen bonding and base-stacking interactions. In fact, base stacking interactions were incorporated in the very first such model in Hyeon and Thirumalai PNAS 2005 and hydrogen bonding was explicitly incorporated later in Denesyuk and Thirumalai, JPCB 2013.

2) The authors note in Discussion and Conclusion section that non-canonical base pairs are not explicitly represented and that this omission is justified empirically but give no reference or further explanation. There really needs to be a much more fuller reasoning and description of what they did. Did non-canonical base pairs really have no hydrogen bonding or base stacking interactions in their simulations? How many non-canonical base pairs are in DDX3X? Local RNA strand displacement often involves non-canonical intermediate states or transient bulge configurations, which would impact their results of partially wound (PU) states.

3) A U-Net architecture is traditionally designed for semantic segmentation of 2D grid image data. The authors demonstrated that Entropy-Unet performs well, as shown in Fig. S5, there appears to be very subtle differences compared to other models. Also, as mentioned in the discussion, 2D convolutions are invariant to translation but biomolecules also require rotational invariance. The authors should describe how they addressed it to prevent artifactual bias.

4) The authors report the previously unrecognized physical mechanism for RNA duplex unwinding. Can the authors also suggest experiments that can check their predictions?

Minor Comments:

1) In abstract (line 28), “dominate factor” should be “dominant factor”.

2) The box sizes should be reported as 30 x 30 x 30 nm3, not 303 nm3.

3) In the funding section, it should read “Ministry of Education of China”, not “Chain”.

4) In Fig. 1d caption, NaCL should be NaCl.

Reviewer #2: For “ATP-Independent Entropy-Driven dsRNA Unwinding by DDX3X Revealed by Coarse-Grained Simulations and Deep Learning” by Wang et al.

The authors have developed a physics-based coarse-grained (CG) RNA model with incorporating weak and specific interactions between DEAD-box RNA helicases (DDX3X) and dsRNA, to explore the inherently stochastic nature of dsRNA unwinding. The work revealed that the dsRNA unwinding process need to overcome an energy barrier, whereas thermal fluctuations and entropy gain provide a driving force for RNA remodeling. Moreover, the work revealed that dsRNA separation proceeds through rare but obligatory strand-displacing intermediates by DDX3X. The work provides a deep understanding on how DDX3X promotes dsRNA unwinding. The manuscript is well-organized. I am pleased to recommend its acceptance on PlosCB after the authors properly address the following issues.

1, The authors proposed a three-bead CG model for dsRNA and validating the model through dsRNA thermal stability and elasticity. The three-bead representation in the model is different from those in existing CG models for RNAs such as SimRNA, Vfold, IsRNA, TiRNA and etc. It would be rather valuable if the authors could add sentences to state the rational for such CG representation and to discuss the possibility to extending the present model to a more complete model for predicting RNA 3D structures or predicting RNA structure stability.

2, I still suggest that the authors can add a brief description for the proposed structure model in the main text although the model is described in details in the Supporting Information. It would be very helpful for PlosCB readers.

3, In SI, the authors described the energy components of their model in details, and there are some parameters for the energy components. The author may need to add a table in the SI to show the values for the parameters, which would be helpful for potential users.

4, The authors proposed an Entropy-UNet method to predict coordination-resolved entropy (CRE) to capture the conformational changes associated with dsRNA unwinding. I am not sure whether the method can be extended or directly applicable for RNA conformation folding. It would be valuable for readers if the authors can clarify the issue, given that the code of Entropy-UNet has been available at github.

5, some very minor issues:

A, In the caption of Fig. 1, the related experimental references may need to be added;

B, In Equation 2, what is the unit of \Delta G? It would be helpful if the authors could explain the coefficient before kBT such as 0.001N_A;

C, A minor inconsistency exists between the main text and supporting information on the corresponding authors.

Reviewer #3: In this manuscript, Wang et al. investigate ATP-independent unwinding of a double-stranded RNA by RNA helicase DDX3X using a new coarse-grained model. The authors validated the model against melting curves of two dsRNAs and then applied it to simulations of DDX3X–dsRNA complex. From simulations, they identify several conformational states and argue that dsRNA unwinding occurs stochastically through thermally driven fluctuations and is primarily entropy-driven. To support this, the authors introduce a coordination-resolved entropy (CRE), defined from the distribution of local coordination numbers, and use this quantity to monitor structural disorder during unwinding. They further develop a deep-learning model, Entropy-UNet, to predict CRE and to decompose the apparent contributions of hydrogen bonding, base stacking, and backbone interactions.

The topic is potentially interesting, and the combination of coarse-grained simulations with ML-based structural analysis could in principle provide useful insight into RNA-protein interactions.

I previously reviewed this manuscript for another journal. Having read it again, I am pleased to see that the authors have revised it in line with the suggestions made by the reviewers, including myself. However, there are still several issues that remain unresolved.

Major points:

(1) The first point remains about the model validity. It remains unclear whether the coarse-grained model can represent general dsRNA properties, particularly unwinding dynamics. I appreciate that the authors have added another dsRNA system, so that two dsRNAs in total are now tested for their ability to reproduce melting temperatures. However, to investigate enzyme-mediated unwinding, particularly when decomposing the contributions of different energetic components such as stacking, hydrogen bonding, and backbone dynamics, as they did in the paper, the model must be validated to ensure that these components are correctly balanced.

Based on the authors' observation of an "open-ring configuration" (line 132), I suspect that the stacking interaction may be overestimated. According to experimental evidence, for example Plumridge et al (2020) 10.1021/jacs.9b04461, single-stranded RNA should not exhibit such a perfectly stacked structure. This may also imply that hydrogen bonding is underestimated in the model (so that melting stability is compensated), which could lead to inaccurate unwinding dynamics. For this specific point, the authors could validate the end-to-end distance and/or persistence length of single-stranded RNA.

In addition, regarding the interaction between RNA and proteins, the authors wrote, (line 163) "We progressively increased the interaction strength between the D1/D2 domains and the Sugar-Phosphate Bead (SPB) in increments of 0.72ε (where ε is the binding energy in kal/mol) until observable dissociation events occurred. This procedure yielded a measured binding energy of 10.00ε between dsRNA and the D1/D2 domains of DDX3X." --- What does this mean? The interaction parameter between the protein and RNA is particularly important in this study, which focuses on binding and helicase activity. Please could you describe in sufficient detail how the 10.00 \epsilon value was obtained? I do not understand the text above. If you increase the strength, don't you observe association events rather than dissociation?

(2) The purpose of developing the deep-learning framework Entropy-UNet is still not clear. In the SI, page 6, it is described as a "specialized statistical framework for predicting and decoupling coordination-resolved entropy." However, the coordination-resolved entropy (CRE) is defined as a function of the coordinates. Why, then, is it necessary to "predict" CRE for a given structure using a neural network? CRE should be directly calculable using Eqs. 3–6 in the main text.

Similarly, for "decoupling", it should also be possible to decompose the coordinate data directly into backbone, HB-bead, and other components. Even more directly, such decomposition could be performed based on the energies, since the coarse-grained model already contains distinct energy terms for stacking, base pairing, and backbone dynamics. I did not fully understand the motivation of using the ML approach here.

(3) How did the authors convert simulation steps to real time? It is claimed that the simulations were performed with a 0.1 ns time step (SI page 5, first paragraph) and accumulated for 100 ms, but if this time step is the actual value used in the numerical integration, the covalent bond potential listed in Table S5 should cause numerical instability. It is often difficult to estimate real-time equivalence in coarse-grained models, particularly when underdamped Langevin dynamics is used. This point should therefore be clarified in the Methods or SI. Alternatively this could be clarified by making LAMMPS input files publicly available.

(4) The coarse-grained simulation code (or complete input for LAMMPS), not only the Entropy-UNet code, should be made publicly available. The analysis code would also be valuable for transferability and reproducibility.

Minor points

- line 47, He et al. (7) - the convention is to use the last name, therefore Song and Ji.

- line 165, kal/mol

- line 520, "Calculate single-bead a mollified version"

**********

**Have the authors made all data and (if applicable) computational code underlying the findings in their manuscript fully available?**

The PLOS Data policy requires authors to make all data and code underlying the findings described in their manuscript fully available without restriction, with rare exception (please refer to the Data Availability Statement in the manuscript PDF file). The data and code should be provided as part of the manuscript or its supporting information, or deposited to a public repository. For example, in addition to summary statistics, the data points behind means, medians and variance measures should be available. If there are restrictions on publicly sharing data or code —e.g. participant privacy or use of data from a third party—those must be specified.

Reviewer #1: None

Reviewer #2: Yes

Reviewer #3: **No:**  The coarse-grained simulation code (or complete input for LAMMPS), not only the Entropy-UNet code, should be made publicly available. The analysis code would also be valuable for transferability and reproducibility.

**********

PLOS authors have the option to publish the peer review history of their article (what does this mean?). If published, this will include your full peer review and any attached files.

If you choose “no”, your identity will remain anonymous but your review may still be made public.

**Do you want your identity to be public for this peer review?**  For information about this choice, including consent withdrawal, please see our Privacy Policy.

Reviewer #1: **Yes:**  Samuel S. Cho

Reviewer #2: No

Reviewer #3: No

[NOTE: If reviewer comments were submitted as an attachment file, they will be attached to this email and accessible via the submission site. Please log into your account, locate the manuscript record, and check for the action link "View Attachments". If this link does not appear, there are no attachment files.]

**Figure resubmission:**

**Reproducibility:**

?>

# Author comment: Author response to Decision Letter 1

**DOI:** 10.1371/journal.pcbi.1014928.r002

16 Sep 2026

Attachment Submitted filename: Response to Reviewers.pdf

# Peer review: Decision Letter 1

**DOI:** 10.1371/journal.pcbi.1014928.r003

**Authors:** Shi-Jie Chen, Dirk Walther

12 Oct 2026

PCOMPBIOL-D-26-01768R1

ATP-Independent Entropy-Driven dsRNA Unwinding by DDX3X Revealed by Coarse-Grained Simulations and Deep Learning

PLOS Computational Biology

Dear Dr. Ren,

Thank you for submitting your manuscript to PLOS Computational Biology. After careful consideration, we feel that it has merit but does not fully meet PLOS Computational Biology's publication criteria as it currently stands. Therefore, we invite you to submit a revised version of the manuscript that addresses the points raised during the review process.

Please submit your revised manuscript by Nov 11 2026 11:59PM. If you will need more time than this to complete your revisions, please reply to this message or contact the journal office at ploscompbiol@plos.org. When you're ready to submit your revision, log on to https://www.editorialmanager.com/pcompbiol/ and select the 'Submissions Needing Revision' folder to locate your manuscript file.

Please include the following items when submitting your revised manuscript:

* A letter that responds to each point raised by the editor and reviewer(s). You should upload this letter as a separate file labeled 'Response to Reviewers'. This file does not need to include responses to formatting updates and technical items listed in the 'Journal Requirements' section below.

* A marked-up copy of your manuscript that highlights changes made to the original version. You should upload this as a separate file labeled 'Revised Manuscript with Track Changes'.

* An unmarked version of your revised paper without tracked changes. You should upload this as a separate file labeled 'Manuscript'.

If you would like to make changes to your financial disclosure, competing interests statement, or data availability statement, please make these updates within the submission form at the time of resubmission. Guidelines for resubmitting your figure files are available below the reviewer comments at the end of this letter.

As the corresponding author, your ORCID iD is verified in the submission system and will appear in the published article. PLOS supports the use of ORCID, and we encourage all coauthors to register for an ORCID iD and use it as well. Please encourage your coauthors to verify their ORCID iD within the submission system before final acceptance, as unverified ORCID iDs will not appear in the published article. Only the individual author can complete the verification step; PLOS staff cannot verify ORCID iDs on behalf of authors.

We look forward to receiving your revised manuscript.

Kind regards,

Shi-Jie Chen

Academic Editor

PLOS Computational Biology

Dirk Walther

Section Editor

PLOS Computational Biology

**Additional Editor Comments (if provided):**

Please see comments by reviewer 1, which I fully endorse.

Best regards,

Dirk Walther

**Reviewers' comments:**

Reviewer's Responses to Questions

**Comments to the Authors:**

**Please note here if the review is uploaded as an attachment.**

Reviewer #1: Overall, I appreciate that the authors provided a detailed and thoughtful rebuttal. The responses provided in the letter adequately addresses my technical and theoretical concerns. However, I noticed that very few of these substantive explanations were incorporated into the revised manuscript itself. Since future readers will likely not have the benefit of reading the rebuttal letter, these excellent clarifications should be integrated directly into the main text or supplementary information. Looking at the other reviewer responses, I imagine that they would feel similarly (especially reviewer #3 who brought up excellent points), but I will focus on my own review here.

1) The authors provide a strong explanation for how their HelixTriad model builds on the three-bead model by decoupling base-stacking and hydrogen bond interactions and sets the foundation for protein-RNA interactions. It seems natural to add this rationale in the introduction or method section to clarify the model’s advancement over previous iterations, namely the Hyeon and Thirumalai, Denesyuk and Thirumalai, and Kapoor, Kim, and Mittal three-bead models.

2) The authors make a fairly bold prediction from their model about the previously unrecognized physical mechanism for RNA duplex unwinding. I think that the suggestions for future experimental validation using single molecule tools like deFCCS and smFRET-anisotropy to detect transient partially unwound intermediates are excellent. It would benefit this manuscript to integrate this discussion into the Discussion or Conclusion to actively guide future in vitro testing.

Reviewer #2: The authors have almost fully addressed my concerns in the last round review. Now I recommend its acceptance.

Reviewer #3: I appreciate that the authors have addressed most of the reviewer comments in the revised manuscript and rebuttal. Although I still have some reservations about the quantitative accuracy of the RNA coarse-grained model, I also acknowledge that the authors have described the model in very detail, and that the simulation code, parameters, and relevant implementation details have been made openly available. Taking these revisions into account, and considering that the manuscript now provides sufficient methodological detail and open resources for readers to assess the validity and limitations of the model, I think the manuscript may be suitable for publication.

However, I would like to record one remaining point of disagreement with the authors’ rebuttal. In response to concerns about the behaviour of single-stranded RNA in their coarse-grained model, the authors compared the end-to-end distance obtained from their coarse-grained simulations with that from Amber atomistic MD simulations. In my view, this comparison does not validate the coarse-grained force field. Atomistic MD simulations should not be treated as a ground truth for RNA conformational ensembles. In particular, it is known that Amber RNA force fields can substantially overestimate compact conformations of single-stranded RNA. For example, see Fig. 3C of Tan et al., PNAS 2018, 10.1073/pnas.1713027115.

This is a common misconception. Atomistic MD simulations are not always correct, particularly for RNA. Since experimental data are available for simple ssRNA systems such as poly(A) and poly(U), validation of single-stranded RNA behaviour should ideally be performed against experimental observables rather than only against Amber simulations.

Although the authors discussed this ssRNA comparison in the rebuttal, the revised manuscript appears to include only a dsRNA comparison and not the ssRNA simulation result. Therefore, I do not request further revision regading this.

**********

**Have the authors made all data and (if applicable) computational code underlying the findings in their manuscript fully available?**

The PLOS Data policy requires authors to make all data and code underlying the findings described in their manuscript fully available without restriction, with rare exception (please refer to the Data Availability Statement in the manuscript PDF file). The data and code should be provided as part of the manuscript or its supporting information, or deposited to a public repository. For example, in addition to summary statistics, the data points behind means, medians and variance measures should be available. If there are restrictions on publicly sharing data or code —e.g. participant privacy or use of data from a third party—those must be specified.

Reviewer #1: Yes

Reviewer #2: Yes

Reviewer #3: Yes

**********

PLOS authors have the option to publish the peer review history of their article (what does this mean?). If published, this will include your full peer review and any attached files.

If you choose “no”, your identity will remain anonymous but your review may still be made public.

**Do you want your identity to be public for this peer review?**  For information about this choice, including consent withdrawal, please see our Privacy Policy.

Reviewer #1: **Yes:**  Samuel S. Cho

Reviewer #2: No

Reviewer #3: No

[NOTE: If reviewer comments were submitted as an attachment file, they will be attached to this email and accessible via the submission site. Please log into your account, locate the manuscript record, and check for the action link "View Attachments". If this link does not appear, there are no attachment files.]

**Figure resubmission:**

After uploading your figures to PLOS’s NAAS tool - https://ngplosjournals.pagemajik.ai/artanalysis, NAAS will process the files provided and display the results in the "Uploaded Files" section of the page as the processing is complete. If the uploaded figures meet our requirements (or NAAS is able to fix the files to meet our requirements), the figure will be marked as "fixed" above. If NAAS is unable to fix the files, a red "failed" label will appear above. When NAAS has confirmed that the figure files meet our requirements, please download the file via the download option, and include these NAAS processed figure files when submitting your revised manuscript.

**Reproducibility:**

To enhance the reproducibility of your results, we recommend that authors of applicable studies deposit laboratory protocols in protocols.io, where a protocol can be assigned its own identifier (DOI) such that it can be cited independently in the future. Additionally, PLOS ONE offers an option to publish peer-reviewed clinical study protocols. Read more information on sharing protocols at https://plos.org/protocols?utm_medium=editorial-email&utm_source=authorletters&utm_campaign=protocols

# Author comment: Author response to Decision Letter 2

**DOI:** 10.1371/journal.pcbi.1014928.r004

14 Oct 2026

Attachment Submitted filename: Response to Reviewers-10-14.pdf

# Editor report: Decision Letter 2

**DOI:** 10.1371/journal.pcbi.1014928.r005

**Authors:** Shi-Jie Chen, Dirk Walther

20 Oct 2026

Dear Dr. Ren,

We are pleased to inform you that your manuscript 'ATP-Independent Entropy-Driven dsRNA Unwinding by DDX3X Revealed by Coarse-Grained Simulations and Deep Learning' has been provisionally accepted for publication in PLOS Computational Biology.

Before your manuscript can be formally accepted you will need to complete some formatting changes, which you will receive in a follow up email. A member of our team will be in touch with a set of requests.

Please note that your manuscript will not be scheduled for publication until you have made the required changes, so a swift response is appreciated.

IMPORTANT: The editorial review process is now complete. PLOS will only permit corrections to spelling, formatting or significant scientific errors from this point onwards. Requests for major changes, or any which affect the scientific understanding of your work, will cause delays to the publication date of your manuscript.

Should you, your institution's press office or the journal office choose to press release your paper, you will automatically be opted out of early publication. We ask that you notify us now if you or your institution is planning to press release the article. All press must be co-ordinated with PLOS.

Thank you again for supporting Open Access publishing; we are looking forward to publishing your work in PLOS Computational Biology.

Best regards,

Shi-Jie Chen

Academic Editor

PLOS Computational Biology

Dirk Walther

Section Editor

PLOS Computational Biology

***********************************************************

# Editor report: Acceptance letter

**DOI:** 10.1371/journal.pcbi.1014928.r006

**Authors:** Shi-Jie Chen, Dirk Walther

PCOMPBIOL-D-26-01768R2

ATP-Independent Entropy-Driven dsRNA Unwinding by DDX3X Revealed by Coarse-Grained Simulations and Deep Learning

Dear Dr Ren,

I am pleased to inform you that your manuscript has been formally accepted for publication in PLOS Computational Biology. Your manuscript is now with our production department and you will be notified of the publication date in due course.

The corresponding author will soon be receiving a typeset proof for review, to ensure errors have not been introduced during production. Please review the PDF proof of your manuscript carefully, as this is the last chance to correct any errors. Please note that major changes, or those which affect the scientific understanding of the work, will likely cause delays to the publication date of your manuscript.

Soon after your final files are uploaded, unless you have opted out, the early version of your manuscript will be published online. The date of the early version will be your article's publication date. The final article will be published to the same URL, and all versions of the paper will be accessible to readers.

For Research, Software, and Methods articles, you will receive an invoice from PLOS for your publication fee after your manuscript has reached the completed accept phase. If you receive an email requesting payment before acceptance or for any other service, this may be a phishing scheme. Learn how to identify phishing emails and protect your accounts at https://explore.plos.org/phishing.

Thank you again for supporting PLOS Computational Biology and open-access publishing. We are looking forward to publishing your work!

With kind regards,

Janani Seenivasan

PLOS Computational Biology | Carlyle House, Carlyle Road, Cambridge CB4 3DN | United Kingdom ploscompbiol@plos.org | Phone +44 (0) 1223-442824 | ploscompbiol.org | @PLOSCompBiol
